# Supplementary material for: A bioinspired hydrogen bond-triggered ultrasensitive ionic mechanoreceptor skin
Source: Nat Commun. 2019 Sep 5;10:4019. doi: 10.1038/s41467-019-11973-5 (PMC6728325; doi:10.1038/s41467-019-11973-5)
Supplement: Supplementary file 1 — Supplementary Information [file 41467_2019_11973_MOESM1_ESM.pdf]

Supplementary Information

A bioinspired hydrogen bond-triggered ultrasensitive ionic mechanoreceptor skin

Amoli *et al*.

List of Contents

Supplementary Note 1: Electrical characterization of SMHIP films

Supplementary Note 2: Development and morphological characterization of various SMHIP films with different ionic liquid content (5–40 wt%)

Supplementary Note 3: Spectroscopic investigation of hydrogen bonding interactions in SMHIP under uniaxial stretching

Supplementary Note 4: Pressure response of different IL-silica-TPU SMHIP films (with varying amount of IL, 5–40 wt%) under different experimental conditions

Supplementary Figures 1 to 24

Supplementary Tables 1 to 9

Supplementary References 1 to 39

Supplementary Note 1: Electrical characterization of SMHIP films

An investigation of the electrical characteristics of the SMHIP films under external pressure can be the key to clarify the pressure-sensing mechanism in SMHIP-based ionic mechanoreceptor skin (Figs. 1e and 1f, Main manuscript). Electrochemical impedance spectroscopy (EIS) is a powerful technique to study ion-transport phenomena in polymer electrolytes and their interfaces (i.e., electrode–electrolyte interfaces). EIS measurements were performed at room temperature using an electrochemical analyzer PGSTAT302N (Metrohm Autolab) in a frequency range of 0.1 Hz to 100 kHz with a 10-mV AC signal. A coin cell assembly provided by Hohsen corp. (Japan) allowed us to perform EIS measurements of different SMHIP films under different experimental conditions (with and without pressure conditions). For EIS measurements, SMHIP films (~170 μm) were sandwiched between two stainless-steel discs (diameter = 10 mm, used as electrodes) to achieve a piezocapacitive device configuration. All of the impedance spectra were fitted using the appropriate equivalent circuit models built in NOVA software (Metrohm Autolab) to evaluate the bulk resistance (R_b) of the devices. The ionic conductivity was calculated from R_b values as follows: $\sigma = \left(\frac{l}{R_b \times A}\right)$, where σ is the ionic conductivity, l is the thickness of polymer film sandwiched between electrodes, A is the area of the electrode, and R_b is the bulk resistance obtained from EIS Nyquist plots.

To support our hypothesis of confined [$EMIM^+$][$TFSI^-$] ion pairs on the surface of silica microstructures in i(20)–silica–TPU SMHIP film, pristine ionic polymer IL(20 wt%)–TPU (i(20)–TPU) as developed in our previous work[1] was also investigated under identical experimental conditions. In i(20)–TPU, most of the ion pairs are mainly located in the free volume derived by TPU soft and hard segments as established by us previously[1]. Bar graph (Supplementary Figure

1a) represents the R_b values for i(20)–silica–TPU SMHIP and i(20)–TPU-based piezocapacitive devices, obtained from the impedance Nyquist plots fitting. Supplementary Figure 1b represents the *ca.* ionic conductivity values. Compared to i(20)–TPU, higher R_b (or lower σ) in i(20)–silica–TPU SMHIP-based piezocapacitive device under no pressure, clearly signifies the confined nature of $[EMIM^+][TFSI^-]$ ion pairs in i(20)–silica–TPU SMHIP film due to the silica microstructures (as confirmed through FE-SEM/EDX elemental analysis, Fig. 2, Main manuscript). The immobilization (or confinement) of $[EMIM^+][TFSI^-]$ ion pairs in i(20)–silica–TPU SMHIP stems from the H-bonding interactions and/or π–π stacking interactions of $[EMIM^+][TFSI^-]$ ion pairs with silica microstructures as confirmed by Raman and Fourier-transform infrared spectroscopy (discussed in molecular characterization section of main manuscript). Similar conclusions have been reported previously for IL–silica ionogels, where interactions between ILs cations and/or anions with silica surface had resulted a greater reduction of ionic mobility[2,3]. Under external pressure, a decrease of R_b (or increase in σ) in i(20)–silica–TPU SMHIP-based piezocapacitive device (similar to i(20)–TPU ionic polymer) reflects the movement of ions under pressure. Interestingly, the high value of ΔR_b ($R_{no\ pressure} - R_{under\ pressure}$) in i(20)–silica–TPU SMHIP (ΔR_b ~204.51 Ω) piezocapacitive device, compared to i(20)–TPU (ΔR_b ~55.79 Ω) signifies a kind of state change of ions i.e., from confined state (under no pressure) to unconfined or bulk-like state (under external pressure) in i(20)–silica–TPU SMHIP. In other words, dramatic increase in σ (or decrease in R_b) in i(20)–silica–TPU SMHIP device clearly reflects the pumping of ions from i(20)–silica–TPU SMHIP film to the i(20)–silica–TPU/electrode interfaces under external pressure. Movement of ions under mechanical pressure is well reported in ionic polymers[4,5]. Impressively, after removing the external pressure, the obtained R_b (or σ) for i(20)–silica–TPU SMHIP-based

piezocapacitive device shows excellent recovery of the initial state, which emphasizes the reversible movement of ions in i(20)–silica–TPU SMHIP film after removing the applied external pressure. As mentioned previously, i(20)–silica–TPU SMHIP film consists of noncovalent organization of $[EMIM^+][TFSI^-]$ ion pairs on the surface of silica microstructures. $TFSI^-$ anions are engaged in H-bonds with the silanol groups, surrounded by $EMIM^+$ cations driven by the Columbic coupling force with the anions together with π–π stacking interaction of imidazolium rings. Therefore, breaking and recreation of these noncovalent interactions i.e., H-bonds and π–π stacking interactions in SMHIP films as reported in various self-healing polymers[6,7] and hydrogel[8,9] is considered as the dominant mechanism for reversible behavior of EIS results obtained here. We performed the FT-IR characterization of IL–silica–TPU SMHIP after application of a wide range of external pressures 50 kPa to 19 MPa (explained in molecular characterization section of the main manuscript), which clearly demonstrates the excellent reversibility of molecular interactions in our rationally designed IL–silica–TPU SMHIP.

Supplementary Figure 1c represents the capacitance measurement results obtained for i(20)-silica-TPU SMHIP-based piezocapacitive devices (ITO/i(20)–silica–TPU/ITO, Supplementary Figure 1c, inset) with and without pressure. Capacitance measurements were performed at room temperature using an Agilent E4980A Precision LCR Meter. Piezocapacitive devices were fabricated by sandwiching the IL–silica–TPU films (film thickness ~170 μm, film area 0.7 cm^2) between two ITO glass electrodes (surface resistance ~10 $\Omega\ sq^{-1}$). The silver wires (Nilaco corp., diameter: 50 μm) were attached to the electrodes for connections with the measuring instrument. We used indium tin oxide (ITO) glass as a model electrode to exclude the effects of the change in interfacial contact area with IL-silica-TPU SMHIP films and deformed electrodes. C_0 and C_p denote the capacitance values without and with pressure (~135 kPa) respectively at an operating

voltage of 1mV @ 20 Hz frequency (Supplementary Figure 1c). An ultra-low value of C_0 (61.4 ± 3.2 pF) in no pressure condition reflects the silica induced confinement effects on $[EMIM^+][TFSI^-]$ ion pairs in i(20)–silica–TPU SMHIP film. Interestingly, a drastic increase in capacitance under pressure (C_p = 85100 ± 1224 pF or 85.1 ± 1.2 nF), ~1385 folds higher than C_0 with characteristic electrical double layer (EDL) phenomena under pressure as confirmed by capacitance vs. applied bias frequency plots (Supplementary Figure 1d) clearly supports the pumping of ionic fluids from i(20)–silica–TPU SMHIP film to i(20)–silica–TPU/ITO interface under external pressure. Electrical characterization results presented in this section clearly support the pressure-induced ion pumping mechanism in SMHIP-based ionic mechanoreceptor skin (Fig. 1e, f, Main manuscript) with excellent recovery of initial state (reversible movement of ions to their original positions) after removal of external stimulus.

Supplementary Note 2: Synthesis and the morphological characterization of various SMHIP films with different ionic liquid content (5–40 wt%)

Preparation of IL–silica–TPU SMHIP films involves three main steps (Supplementary Figure 2): (i) Preparation of IL–silica gel through sol-gel; (ii) Preparation of thermoplastic polyurethane (TPU) precursor gel; (iii) Preparation of IL–silica–thermoplastic polyurethane tri-component solution, followed by an optimized heat-treatment process to develop IL–silica–TPU SMHIP films. Supplementary Figure 2 demonstrates the schematic illustration of IL–silica–TPU SMHIP synthesis process under optimized reaction conditions. The reaction mechanism or the nature of interactions among different species at different stages of synthesis procedure can be explained on the basis of the conclusions drawn in previous reports dealing with silica-based materials and/or silica–ionogels prepared under acidic conditions similar to our case[10,11]. Since TEOS is known to

be less sensitive to hydrolysis[11], when HCl is added to the TEOS+H_2O+IL solution (Step-I), hydrolysis-condensation reactions of TEOS start instantaneously, leading to the formation of the silica network (Si-O-Si) with dominant surface Si-OH groups (a well-known reaction mechanism of silica-based materials prepared under acidic conditions[11,12]. At the same moment, IL ($[EMIM^+][TFSI^-]$ ion pairs) interactions with the growing silica network can be explained on the basis of isoelectric point consideration[10]. In our case, the measured *pH* value of the solution becomes 1.5 (at the moment of HCl addition), which is lower than the isoelectric point of silica (2.2)[10], hence silica species get a net positive charge in the solution that can facilitate $TFSI^-$ anion to interact with silica matrix through electrostatic and/or H-bonds interactions.[10] We performed Fourier-transform infrared (FT-IR) spectroscopy analysis of IL–silica gel obtained in step–I. Shifting of $TFSI^-$ FT-IR stretching bands (Supplementary Figure 3a, b) towards lower–wavenumber side in IL–silica gel (compared to pure IL), in agreement with previous reports[2,13], confirms the H-bonding interactions of $TFSI^-$ and the silica surface silanol groups (Supplementary Figure 3c). TPUs are multiblock copolymers with an alternating sequence of hard and soft segments (step–II). In the final step (step–III), when IL–silica gel is mixed with TPU gel under mechanical stirring, the compatibilizing effect of $[TFSI^-][EMIM^+]$[14,15] can facilitate the uniform dispersion of IL–silica species in TPU polymer matrix, which grow as IL–silica microstructures well dispersed in TPU polymeric matrix after an optimized heat treatment process in our work. IL–silica microstructures (dispersed phase) and TPU matrix (continuous phase) correspond to the physical analogues of the living cells and the extracellular matrix of biological multicellular structures.

In our work, 20 wt% of IL is the optimized concentration to obtain shape and size controlled ionic liquid–confined silica structures well dispersed in TPU polymer matrix, as observed for i(20)–

silica–TPU SMHIP (see FE-SEM image, Fig. 2a, Main manuscript). For other concentrations of IL e.g., 5, 10 and 40 wt% termed as i(5)–silica–TPU, i(10)–silica–TPU and i(40)–silica–TPU, uncontrolled silica structures both in sizes and shapes are produced as seen in FESEM images of i(5)–silica–TPU (Supplementary Figure 4a), i(10)–silica–TPU (Supplementary Figure 4b) and i(40)–silica–TPU (Supplementary Figure 4c). As the ratio of IL/TEOS decreases from i(40)–silica–TPU → i(5)–silica–TPU, size of silica structures is increased which clearly indicates that ionic liquid acts as a morphology directing agent for IL–silica structures as reported previously[16]. For an optimized IL and silica precursor ratio (IL/TEOS), effective synergy is expected between ionic species and silica nuclei during growth[14,16] that can result in the formation of well defined, size and shape controlled IL confined silica structures dispersed in TPU polymer matrix, as observed for i(20)–silica–TPU SMHIP (see FESEM image, Fig. 2a, Main manuscript). Despite irregular and uncontrolled IL–silica structures obtained for 5, 10 and 40 wt% of IL content in IL–silica TPU SMHIPs, corresponding elemental analyses of i(5)–silica–TPU (Supplementary Figure 5), i(10)–silica–TPU (Supplementary Figure 6) and i(40)–silica–TPU (Supplementary Figure 7) SMHIP films indicate that synthetic multicellular morphology (where IL–silica microstructures as dispersed phase and TPU matrix as continuous phase correspond to physical analogues of living cells and extracellular matrix of biological multicellular structure respectively as demonstrated in Fig. 1, Main manuscript) is still retained for 5–40 wt% of IL content (Supplementary Figures 5–7).

Supplementary Note 3: Spectroscopic investigation of hydrogen bonding interactions in SMHIP under uniaxial stretching

In order to study nature of interactions in IL-silica-TPU films under external deformation, FT-IR studies of i(20)-silica-TPU SMHIP film during uniaxial stretching at ambient conditions were performed. Supplementary Figure 10 shows the FT-IR spectra of i(20)-silica-TPU SMHIP at normal state (ε (%) = 0) and under strain (ε_s (%) = 50) conditions. In order to clarify the breaking of H-bonds between in SMHIP polymer film, ionic liquid (IL) characteristics region (1400–1000 cm^{-1}, assigned to $TFSI^-$ vibrational bands) and 3210–3060 $cm^{-1,}$ assigned to $EMIM^+$ vibrational bands) and TPU characteristics region (1760–1660 cm^{-1}, assigned to TPU C=O stretching) are investigated. Supplementary Figure 10a shows the FT-IR characteristic regions of $TFSI^-$ and $EMIM^+$ of i(20)-silica-TPU SMHIP at normal state (ε (%) = 0) and under external strain (ε_s (%) = 50). It is important to note that most of the vibrational bands of $TFSI^-$ (SO_2, CF_3, and S–N–S stretching) are shifted to higher frequencies under external strain. The shifting of $TFSI^-$ vibrational bands towards higher frequencies is a reflection of bulk-like dynamics[13,17]. Similarly, the vibrational bands of $EMIM^+$ (C–H symmetric/asymmetric stretch, ring NC(H)NCH stretch, and CH_3(N)HCH stretch) are shifted to higher frequencies under strain. These results obtained here are in excellent agreements with the previous studies dealing with spectroscopic investigations of polymeric materials under external deformations, given that the perturbations to the polymeric matrix caused by external deformations (pressure/strain) are reflected by the shifts of the IR bands.[18,19] Hence, the spectroscopic results obtained for SMHIP films under external strain can be correlated to the weakening/breaking of TFSI/silica H-bonds and/or π–πnce, the spectroscopic reimidazolium rings in SMHIP under external strain. Supplementary Figure 10b represents FT-IR spectra of C=O stretching region of TPU (where the two peaks at ~1729 cm^{-1} and ~1703 cm^{-1} correspond to the stretching vibration of free C=O and hydrogen-bonded C=O groups) of i(20)-silica-TPU SMHIP at normal state (ε (%) = 0) and under strain (ε_s (%) = 50). It is important to note

that the intensity of hydrogen-bonded C=O groups is decreased significantly under strain while the intensity of free C=O is increased, which clearly reflects perturbations in SMHIP H-bond networks. We calculated H-bond index (ratio of the peak area of H-bonded C=O groups/free C=O groups of TPU) of i(20)-silica-TPU SMHIP film in the stretched state and compared with the upstretched condition. A pronounced decrease of ~35.7% in *ca*. H-bond index in the stretched state clearly signifies the breaking/weakening of H-bonds networks in SMHIP under external deformation. The spectroscopic investigation of SMHIP during strain clearly indicates the deformation of H-bond networks under external deformation.

Supplementary Note 4: Pressure response of different IL-silica-TPU SMHIP films (with varying amount of IL, 5–40 wt%) under different experimental conditions

Supplementary Figures 15–18 represent the pressure response of different IL-silica-TPU (5–40 wt% IL) SMHIP-based piezocapacitive pressure sensors, under different applied bias voltages and bias frequencies. Pressure response of all the IL–silica–TPU pressure sensors shows similar trend where continuous increase in the external pressure results in monotonic increase in the capacitance of the device. This can be explained by pressure-induced enhanced ion pumping in IL–silica–TPU SMHIP films and resulting in the increase in the number of ion pairs forming EDL at IL–silica–TPU/ITO electrode interfaces. In other words, it reflects the facile breaking of non-covalent [$EMIM^+$][$TFSI^-$]–silica H-bonds and/or π–π interactions with increased pressure as reported in various supramolecular polymeric network[6,7] and self-healing hydrogels previously[8,9]. Supplementary Figure 19 shows the effect of bias voltage on the initial (C_0, capacitance under no pressure) and the final capacitance (C_p, capacitance under a pressure of ~135 kPa) of i(20)–silica–TPU-based piezocapacitive device. Voltage dependent increasing trends of C_0 and C_p signify that enhancement in electric field across i(20)–silica–TPU affects the capacitance of the device. This

can be explained by weakening of $[EMIM^+][TFSI^-]$–silica H-bonds and/or π–π interactions with increase in applied voltage[20,21] and subsequent voltage driven facile migration of ions[4,5] towards the respective electrodes to strengthen the EDL mechanism. Pressure vs. capacitance curves under different applied bias voltage conditions (Supplementary Figures 15–18) undoubtly prove that the pressure sensing mechanism in SMHIP pressure sensors stems from the pumping of $[EMIM^+][TFSI^-]$ ion pairs from IL–silica–TPU SMHIP to IL–silica–TPU/electrode interfaces. The pressure sensitivities of different IL–silica–TPU SMHIP pressure sensors obtained from their pressure response curves under different applied bias voltages and bias frequencies (Supplementary Figures 15–18) is given in Supplementary Tables 3, 5–7. The pressure sensitivity ($S = \frac{\delta(\Delta C/C_0)}{\delta P}$) of the devices in different pressure regimes was obtained from the slope of the relative change in capacitance versus the pressure. It is important to note that pressure sensitivity values obtained for i(40)–silica–TPU pressure sensor are significantly lower than those of IL–silica–TPU pressure sensors with 10 and 20 wt% of IL, which can be attributed to its relatively low C_p/C_0 (~68) (Supplementary Table 4), which stems from the unconstrained features of $[EMIM^+][TFSI^-]$ ion pairs in i(40)–silica–TPU SMHIP film compared to i(20)–silica–TPU (C_p/C_0~1045) and i(10)–silica–TPU (C_p/C_0~121), since pressure sensitivity $S = \frac{\delta(\Delta C/C_0)}{\delta P}$ is directly proportional to C_p/C_0. The unconstrained features of $[EMIM^+][TFSI^-]$ ion pairs in i(40)–silica–TPU are reflected in XRD results (Fig. 4d, Main manuscript) where increasing IL content is appeared in the intercalation of ions in TPU polymer chains similar to IL–TPU ionic polymers (where most of the ions are located in the free volume derived from TPU hard and soft segments)[1]. For fixed amount of silica, further increase in IL content (beyond 20 wt% in our case) will increase the fraction of ionic liquid that is not bound to silica surface and exhibit bulk-like dynamics[17]

which gives rise to high C_0 (3.99 ± 0.06 nF, Supplementary Table 4) in i(40)–silica–TPU-based piezocapacitive device due to unconstrained movement (bulk like behavior) of unconfined ions towards i(40)–silica–TPU /electrode interface and formation of EDL under an extremely low voltage (1 mV), even under no external pressure condition.

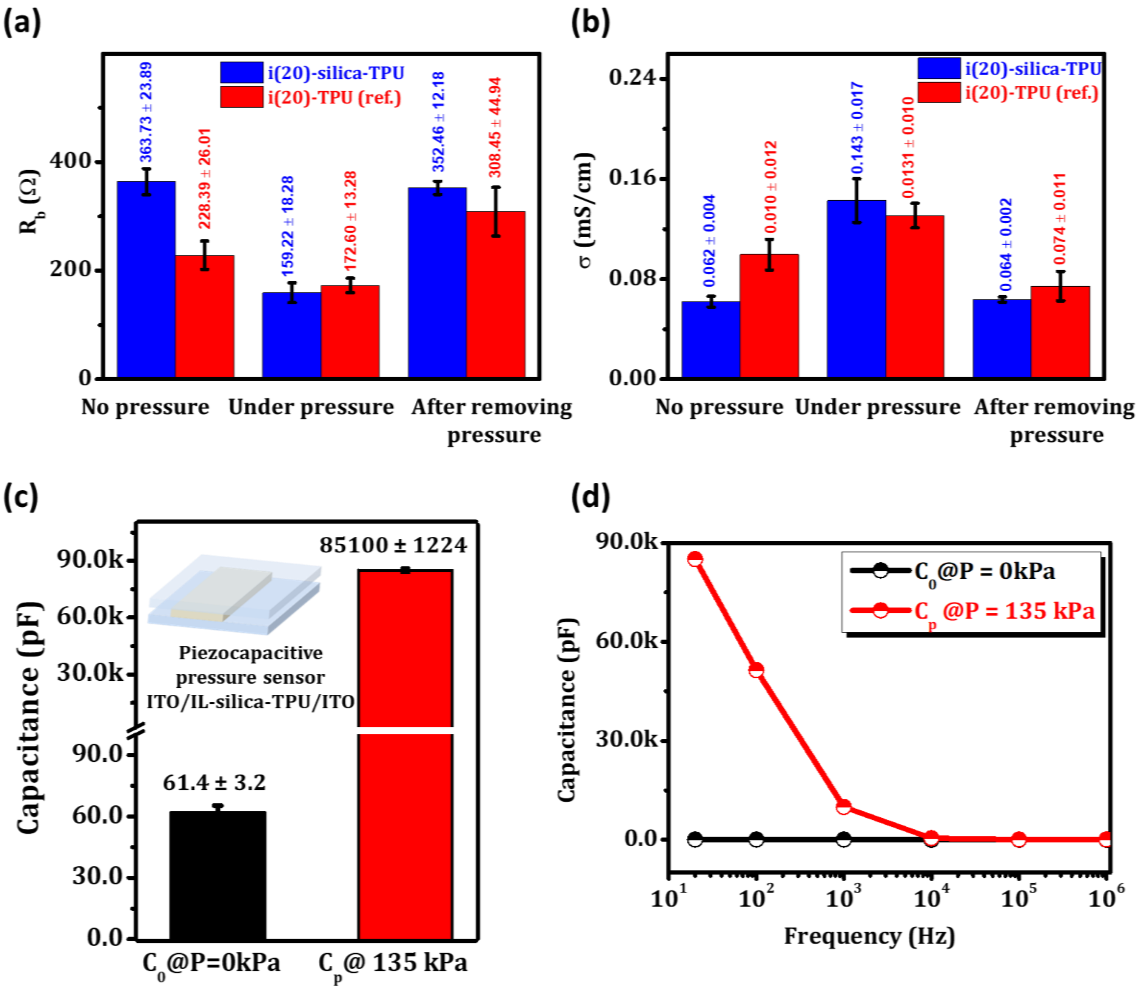

Supplementary Figure 1. Electrical characterization of i(20)–silica–TPU SMHIP. (a) Bar graph represents the bulk resistance (R_b) values obtained from the impedance Nyquist plots of i(20)–silica–TPU SMHIP and i(20)–TPU ionic polymer piezocapacitive devices under no pressure, under external pressure and after removing external pressure. **(b)** Bar graph represents the *ca.* ion conductivity of i(20)–silica–TPU SMHIP and i(20)-TPU ionic polymer piezocapacitive devices under no pressure, under external pressure and after removing external pressure conditions. Error bars represent standard deviation in n = 5 samples. **(c)** Bar graph represents the initial (C_0) and the final capacitance (C_p) capacitance of i(20)–silica–TPU SMHIP piezocapacitive device; C_0 denotes the capacitance without pressure and C_p denotes the capacitance under a pressure of ~135 kPa (1 mV applied bias @ 20 Hz), inset is the schematic of i(20)–silica–TPU piezocapacitive device. Error bars represent standard deviation in n = 5 samples. **(d)** Frequency vs. capacitance plots of C_0 and C_p in i(20)–silica–TPU SMHIP piezocapacitive device.

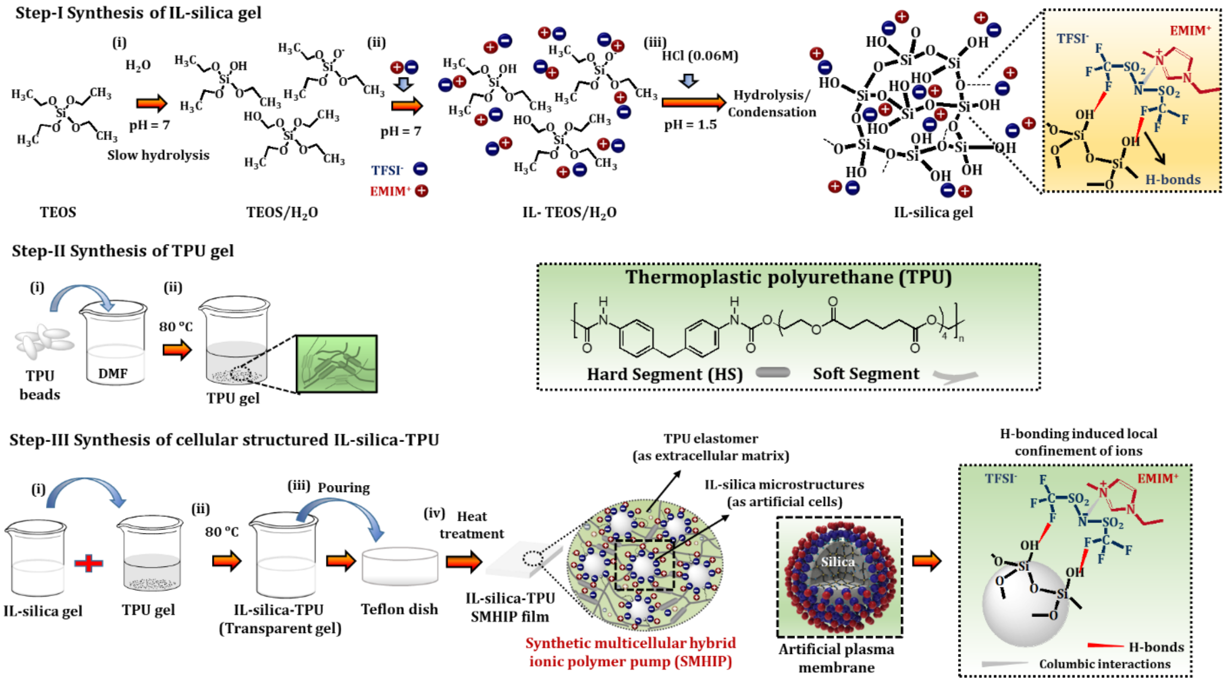

Supplementary Figure 2. Schematic of the synthesis process of IL–silica–TPU SMHIP films. Step–I includes the synthesis of ionic liquid–silica gel under optimized conditions using Tetraethyl orthosilicate (TEOS) as silica precursor and 1-Ethyl-3-methylimidazolium bis(trifluoromethanesulfonyl)imide ([EMIM$^+$][TFSI$^-$]) as ionic liquid (IL) and 0.06 M HCl as an acid catalyst. Step–II includes the preparation of thermoplastic polyurethane (TPU) gel using Dimethylformamide (DMF) as solvent. Step–III includes the preparation of tri-component gel precursor (IL–silica gel + TPU + DMF) followed by casting and heat treatment processes to develop IL–confined silica–TPU SMHIP film. IL–silica microstructures (dispersed phase) and TPU matrix (continuous phase) correspond to physical analogues of the living cell and the extracellular matrix of biological multicellular structure. Molecular characterization results reveal that TFSI$^-$ anions (blue) tethered on the surface of silica microspheres (grey sphere) due to H-bonds interactions with silanol groups, surrounded by EMIM$^+$ cations (red) driven by the Columbic coupling force with the anions together with π–π stacking interaction of imidazolium rings, which constitutes an artificial plasma membrane structure (inset, black dotted box).

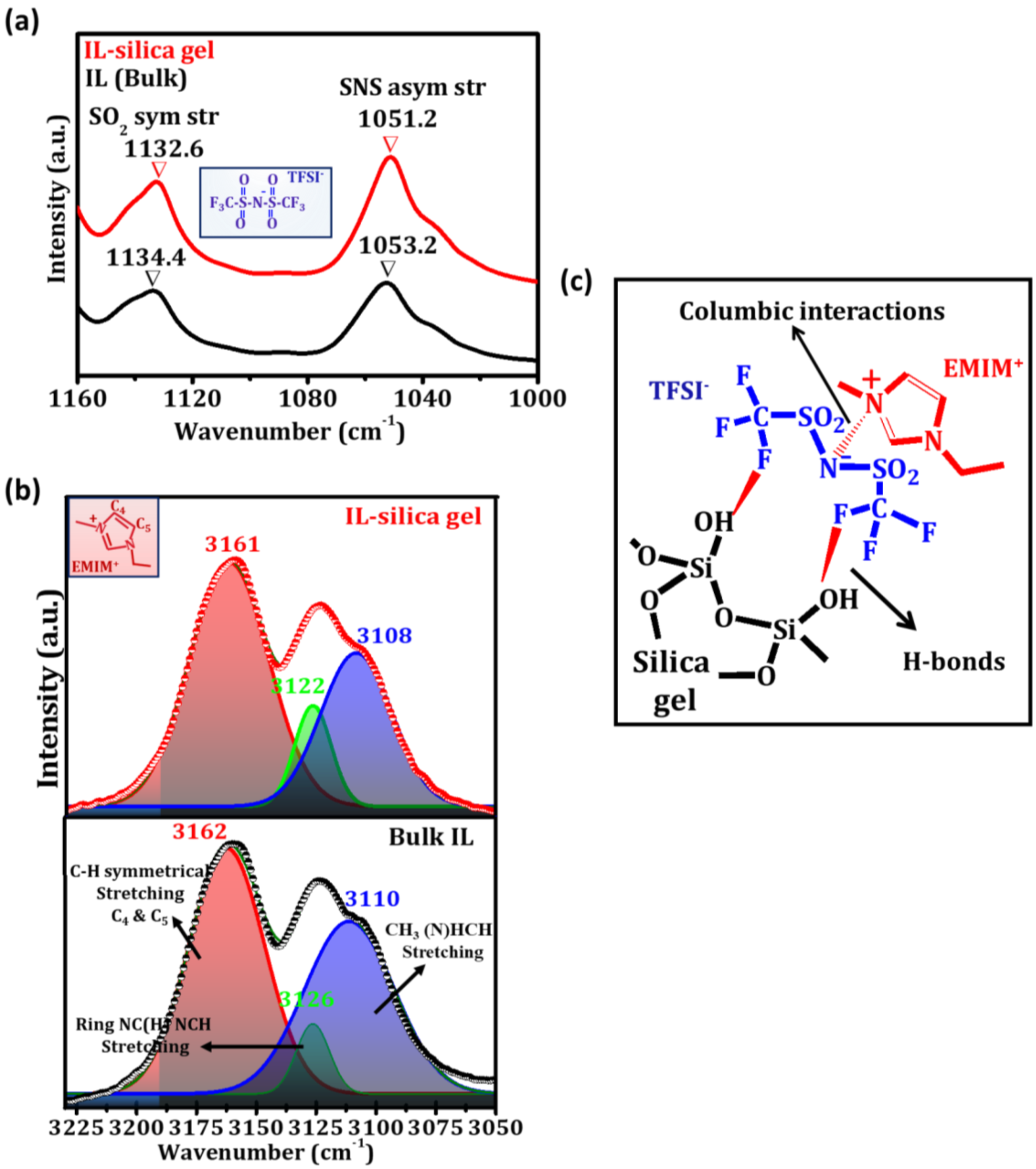

Supplementary Figure 3. FT-IR analysis of [EMIM$^+$][TFSI$^-$] and [EMIM$^+$][TFSI$^-$]–silica gel. **(a)** FTIR spectra in the spectral range 1160–1000 cm^{-1}**,** corresponding to TFSI$^-$ stretching region. Inset is the schematic representation of TFSI$^-$ ion. **(b)** FT-IR spectra in the spectral range 3225–3050 cm^{-1}**,** corresponding to EMIM$^+$ stretching region. Inset is the schematic representation of EMIM$^+$ ion. **(c)** Schematic representation of H-bonding interactions between TFSI$^-$ and the silica surface silanol groups.

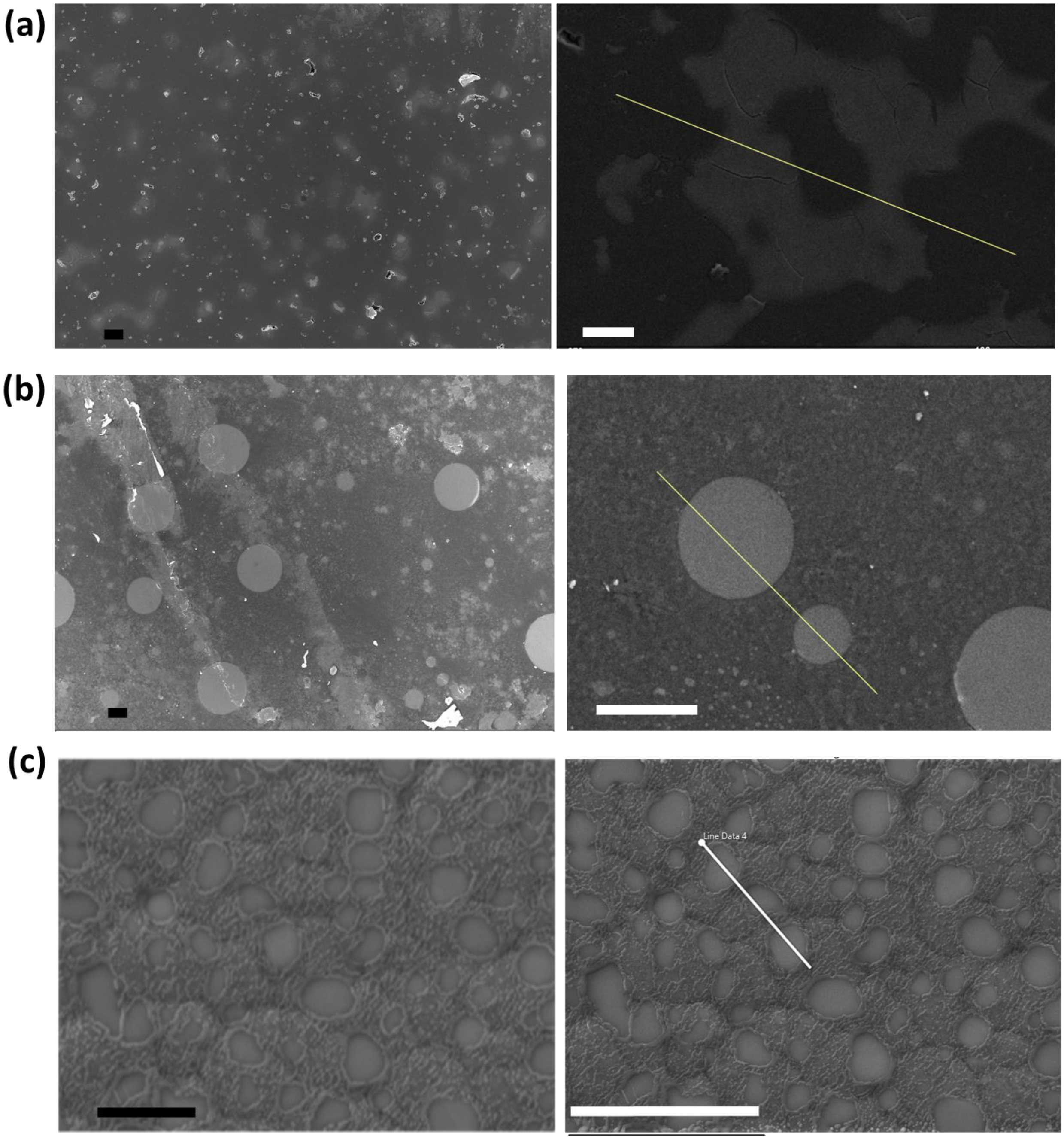

Supplementary Figure 4. FE-SEM images of IL-silica-TPU SMHIP films (5–40 wt%). (a) IL(5 wt%)–silica–TPU (i(5)–silica–TPU) SMHIP. **(b)** IL(10 wt%–silica–TPU (i(10)–silica–TPU) SMHIP. **(c)** IL(40 wt%)–silica–TPU (i(40)–silica–TPU) SMHIP. Scale bars, 20 µm (black); 50 µm (white).

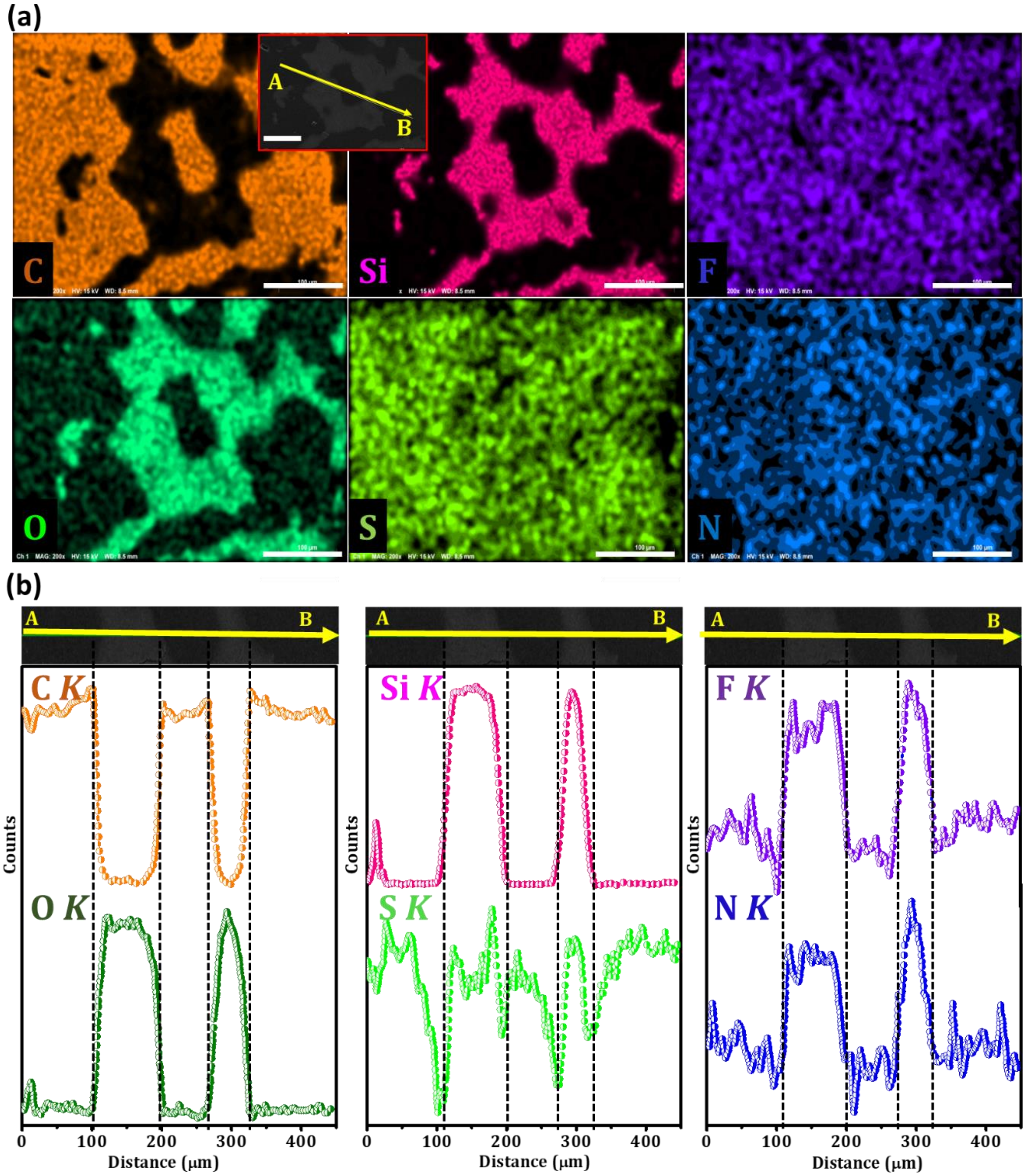

Supplementary Figure 5. FE-SEM/EDX analysis of i(5)–silica–TPU SMHIP. (a) Elemental maps (corresponding to FE-SEM image shown in the inset, red box) for C, Si, F (coming from $TFSI^-$ anion), O, S, and N (coming from $EMIM^+$ cation, $TFSI^-$ anion and TPU collectively); all scale bar 100 μm. Scale bars, 100 μm. **(b)** EDX line scanning profiles along the noted line AB (yellow) as represented in inset for C, Si, F (coming from $TFSI^-$ anion), O, S, and N (coming from $EMIM^+$ cation, $TFSI^-$ anion and TPU collectively). Both the point area elemental maps and line scanning profiles indicate that the dispersed phase is [$EMIM^+$][$TFSI^-$]–silica and the continuous phase is TPU elastomer.

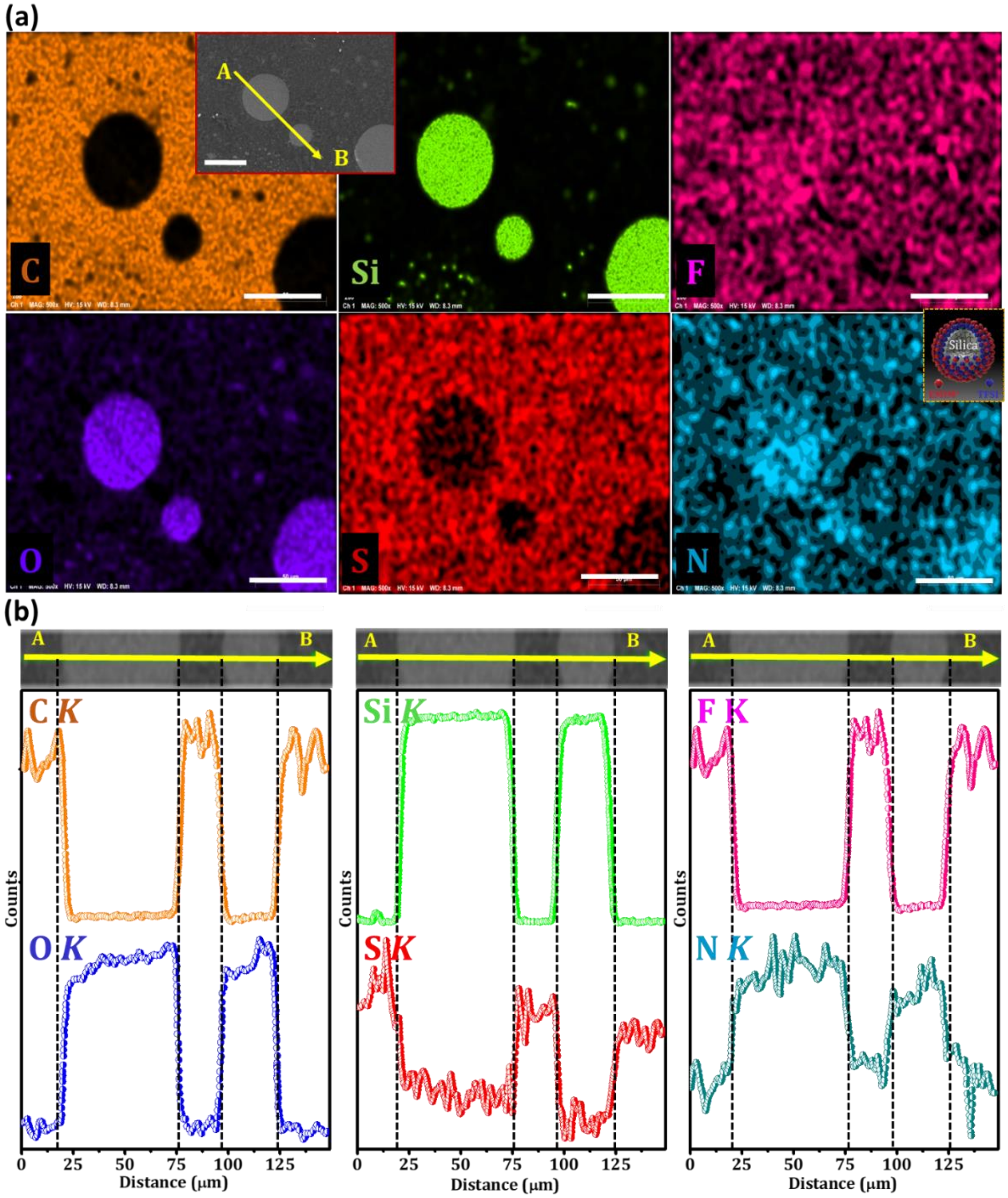

Supplementary Figure 6. FE-SEM/EDX analysis of i(10)–silica–TPU SMHIP. (a) Elemental maps (corresponding to FE-SEM image shown in the inset, red box) for C, Si, F (coming from $TFSI^-$ anion), O, S, and N (coming from $EMIM^+$ cation, $TFSI^-$ anion and TPU collectively). Scale bars, 50 μm. **(b)** EDX line scanning profiles along the noted line AB (yellow) as represented in the inset for C, Si, F (coming from $TFSI^-$ anion), O, S, and N (coming from $EMIM^+$ cation, $TFSI^-$ anion and TPU collectively). High intensities of F and N in the circumferential and inner regions of silica microstructures respectively, establish a microscopic artificial plasma membrane structure consisting of ordered step-wise layers of $TFSI^-$ and $EMIM^+$ on silica microspheres (inset, yellow box, where grey sphere represents silica microstructures, blue anions represent $TFSI^-$ ions and red cations represent $EMIM^+$ ions).

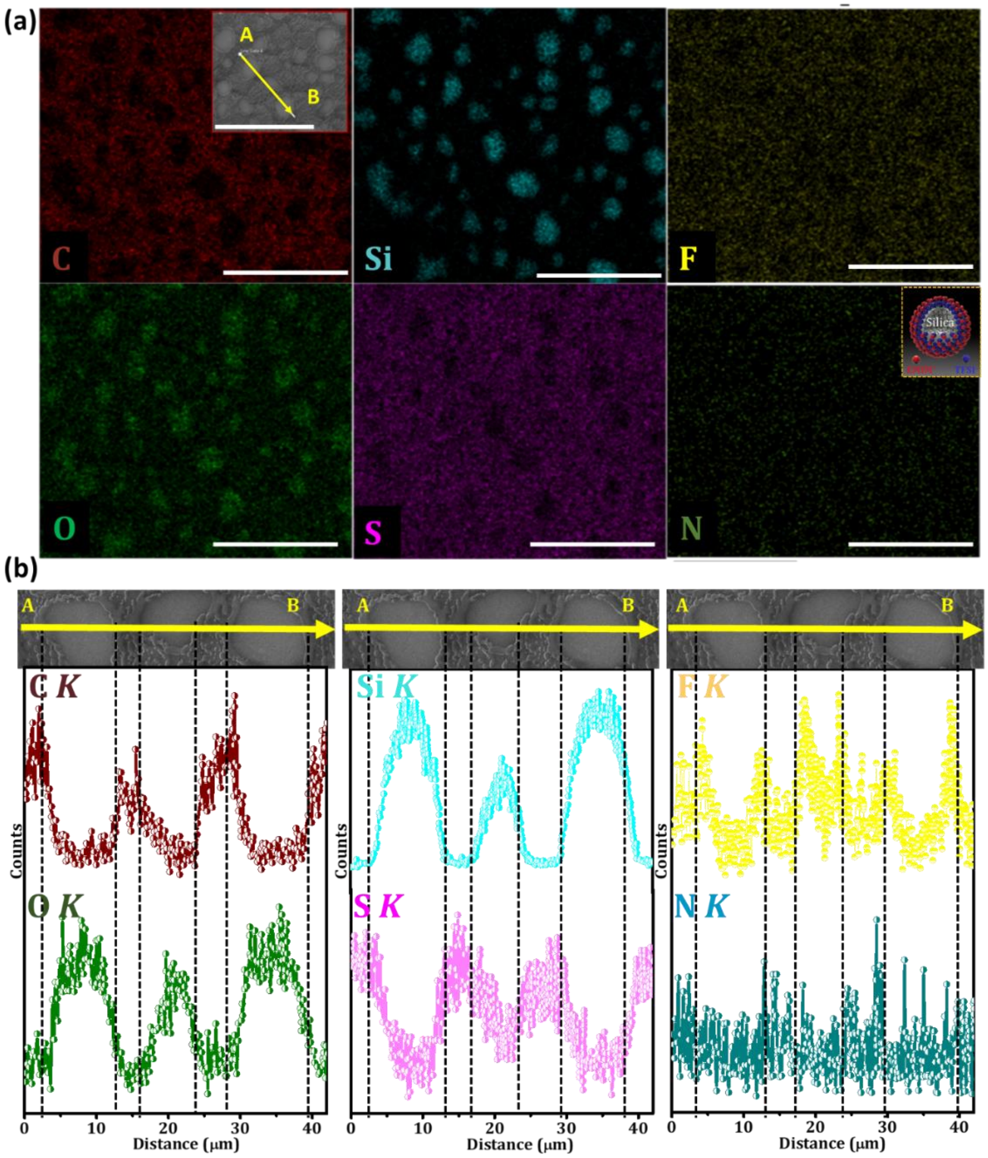

Supplementary Figure 7. FE-SEM/EDX analysis of i(40)–silica–TPU SMHIP. (a) Elemental maps (corresponding to FE-SEM image shown in the inset, red color) for C, Si, F (coming from $TFSI^-$ anion), O, S, and N (coming from $EMIM^+$ cation, $TFSI^-$ anion and TPU collectively). Scale bars, 50 μm. **(b)** EDX line scanning profiles along the noted line AB (yellow) represented in the inset for C, Si, F (coming from $TFSI^-$ anion), O, S, and N (coming from $EMIM^+$ cation, $TFSI^-$ anion and TPU collectively). High intensity of F and N in the circumferential and inner regions of silica microstructures respectively, establish a microscopic artificial plasma membrane structure consisting of ordered step-wise layers of $TFSI^-$ and $EMIM^+$ on silica microspheres (inset yellow box, where grey sphere represents silica microstructures, blue anions represent $TFSI^-$ ions and red cations represent $EMIM^+$ ions).

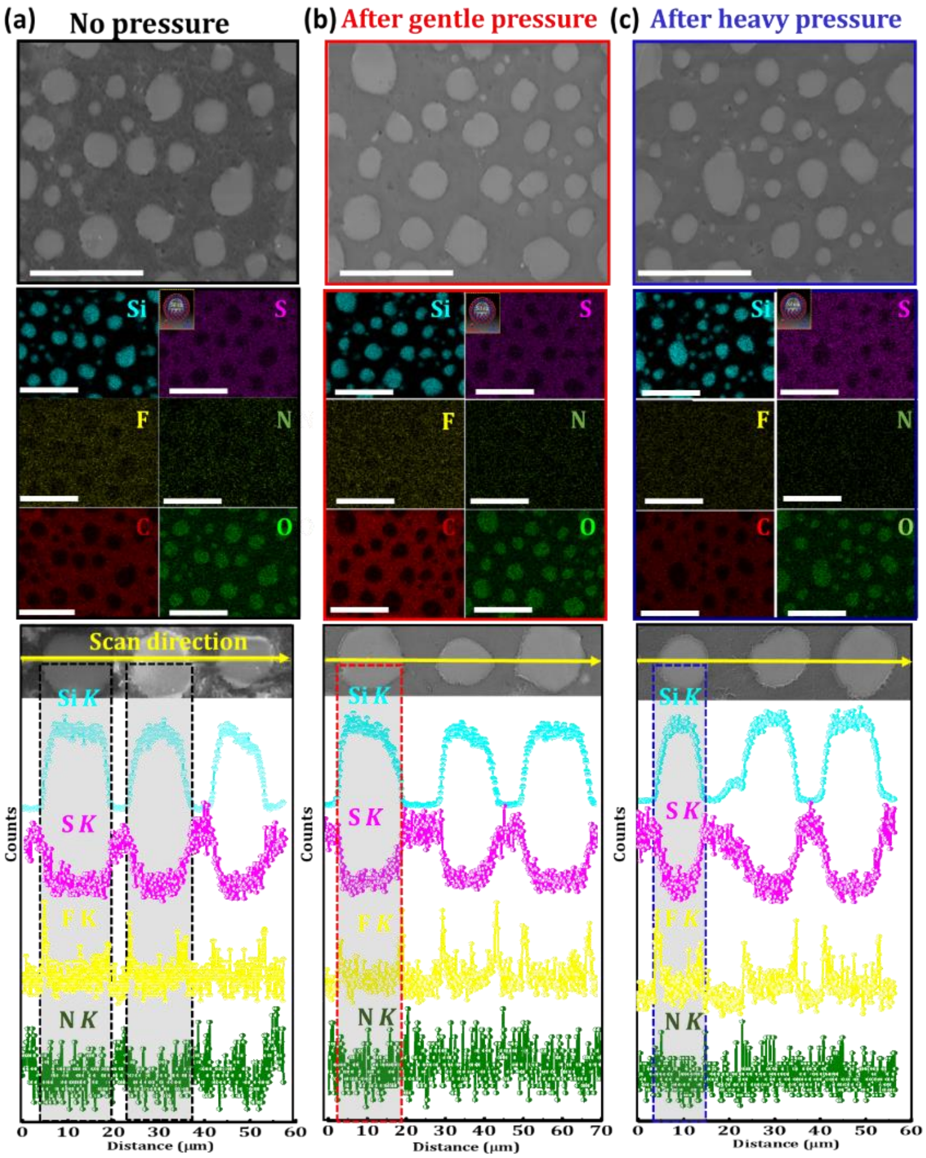

Supplementary Figure 8. FE-SEM/EDX after 10 loading/unloading cycles of mechanical forces. (a) Pristine i(20)–silica–TPU SMHIP. **(b)** After 10 repeated loading/unloading cycles of gentle pressure applied by thumb. **(c)** After 10 repeated loading/unloading cycles of a heavy pressure applied by thumb. All Scale bars, 50 µm. No detectable changes in the morphology and/or elemental distribution are obtained even after application of a wide range of pressures, which reveals the excellent structural integrity of i(20)–silica–TPU SMHIP. High intensity of F and N in the circumferential and inner regions of silica microstructures respectively as detected in Elemental maps/EDX line scans of pristine i(20)–silica–TPU SMHIP (Supplementary Figure 8a) is retained even after application of various repeated loading/unloading cycles of gentle pressure (Elemental maps/EDX line scans, Supplementary Figure 8b) and heavy pressure (Elemental maps/EDX line scans, Supplementary Figure 8c). Inset (yellow box) is the schematic of artificial plasma membrane consisting of ordered step-wise layers of $TFSI^-$ (represented by blue anions) and $EMIM^+$ (represented by red cations) on silica microspheres (represented by grey sphere) based on elemental analysis, which is retained even after the application of a wide range of pressures.

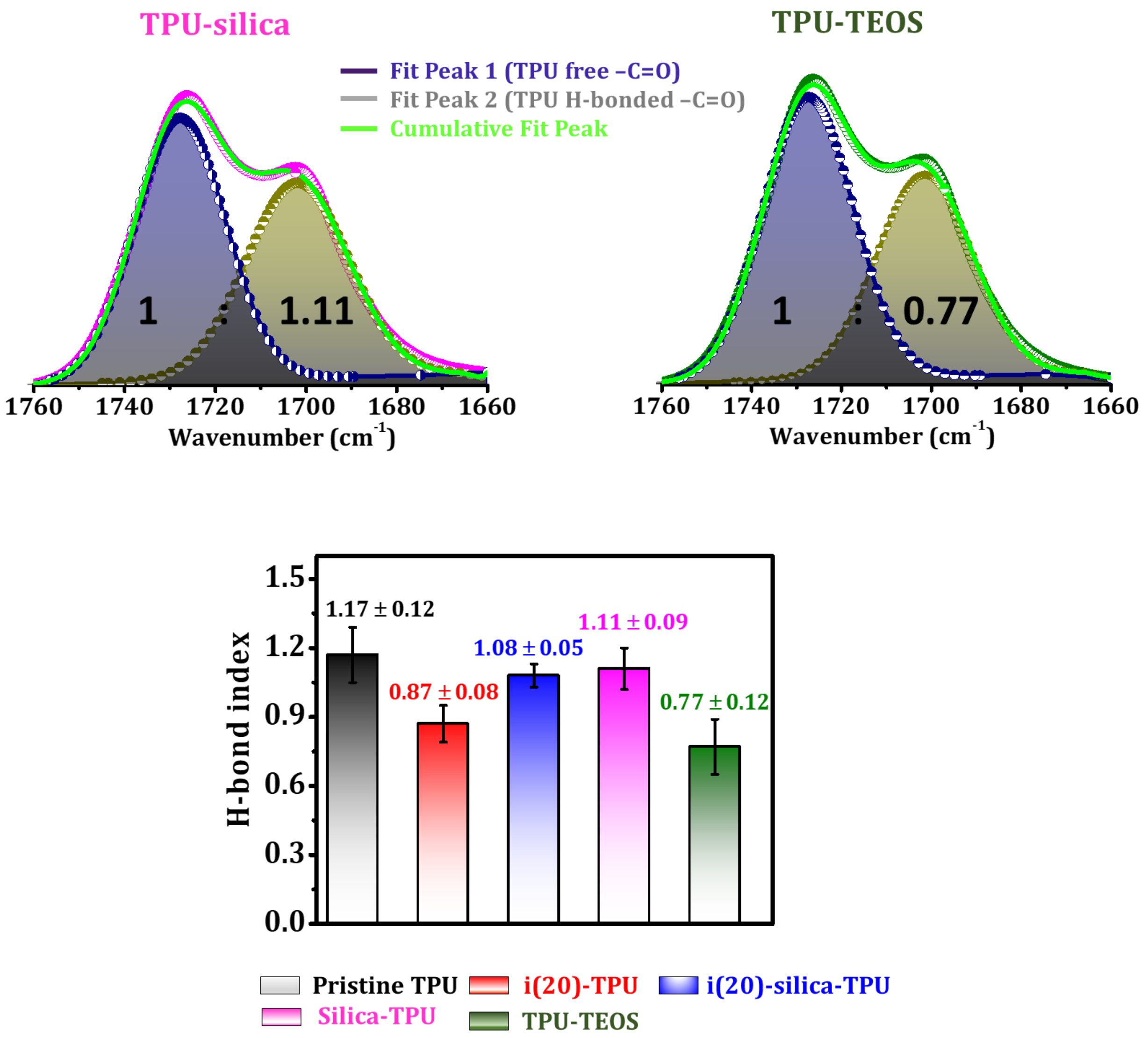

Supplementary Figure 9. FT-IR spectra of TPU-silica and TPU-TEOS composites. ATR-FTIR spectra in the spectral region 1760–1660 cm^{-1} (C=O stretching of TPU). Deconvolution of the FTIR peaks was performed by considering peaks Gaussian with a number of iterations to obtain the best-fit Gaussian peak. The ratio of the peak area of H-bonded C=O bands (centers at approximately 1703 cm^{-1} to the free C=O bands (centers at approximately 1730 cm^{-1}) is termed as H-bond index. Bar graph represents H-bond indices in different composites. Error bars represent standard deviation in n = 5 samples.

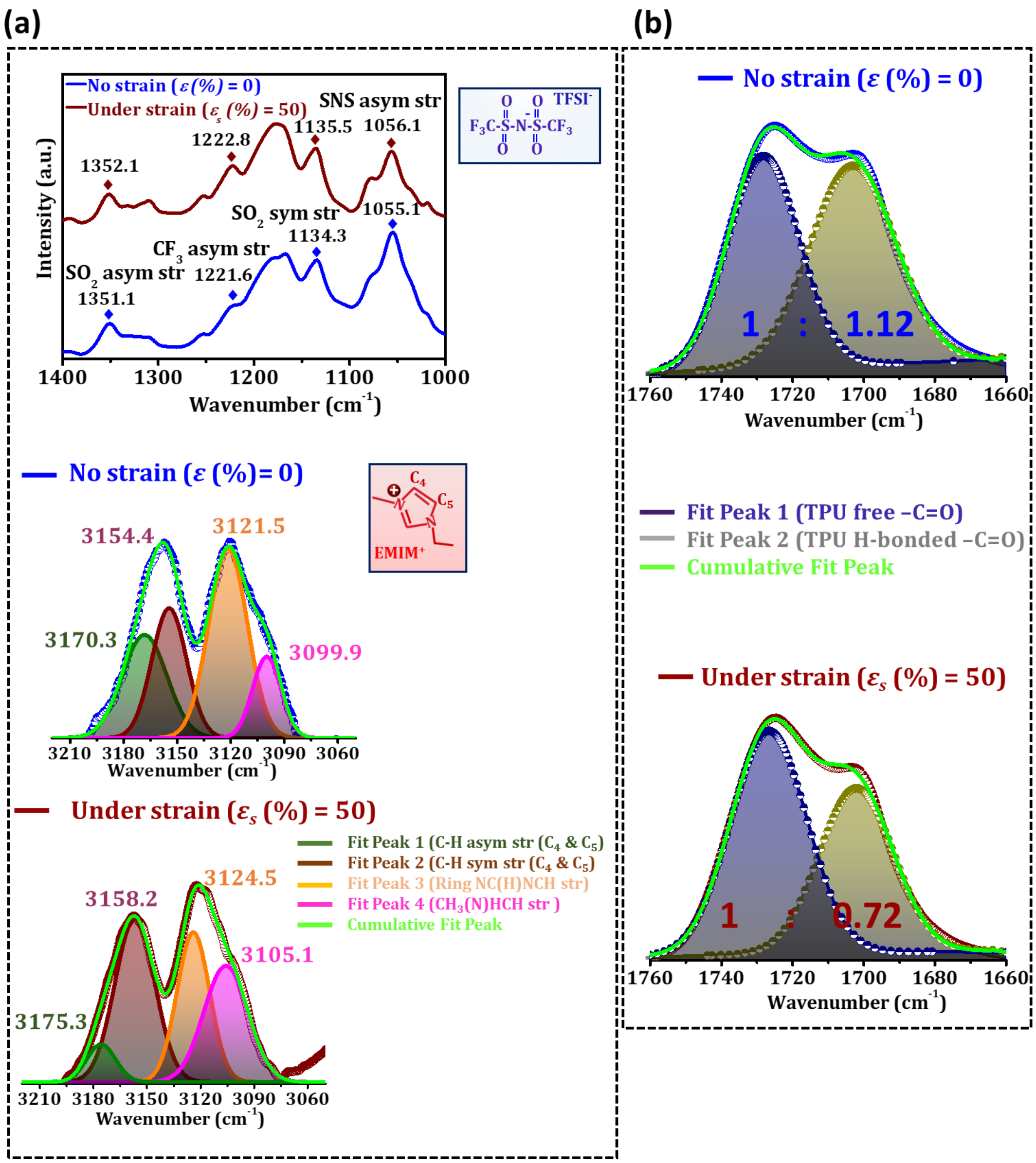

Supplementary Figure 10. FT-IR investigation of i(20)-silica-TPU under uniaxial stretching . (a) ATR-FTIR spectra in the spectral regions 1400–1000 cm^{-1} (corresponding to TFSI$^-$ stretching) and 3210–3060 cm^{-1} (corresponding to EMIM$^+$ stretching); Results are shown for i(20)–silica–TPU SMHIP in normal state (no strain, ε (%) = 0) (shown by blue) and during uniaxial strain (ε_s (%) = 50) condition (shown by red color). **(b)** ATR-FTIR spectra in the spectral region 1760–1660 cm^{-1} (C=O stretching of TPU) in normal state (no strain, ε (%) = 0) (shown by black color) and during strain (ε_s (%) = 50) condition (shown by dark red). Source data for panel **a, b** are provided as a Source data file.

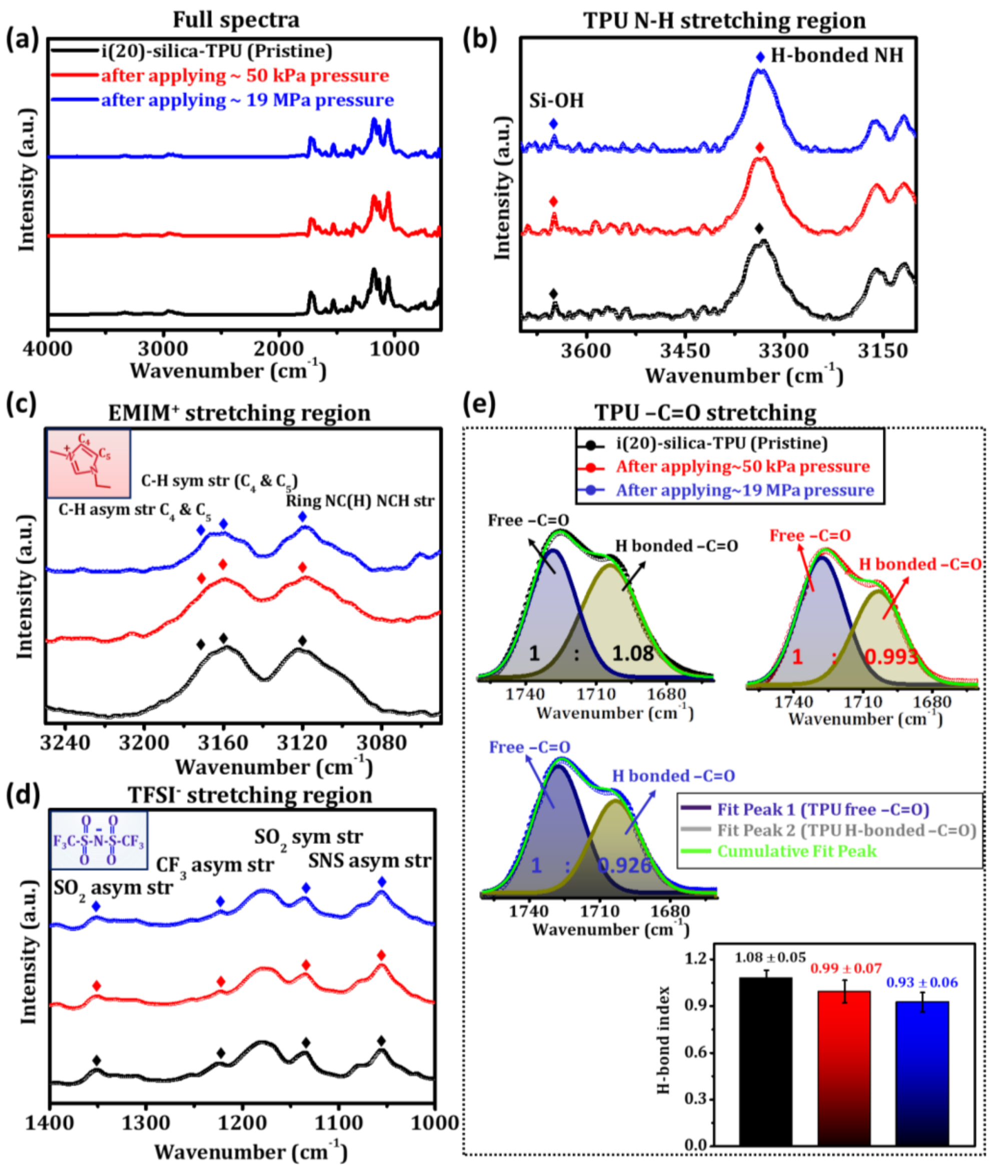

Supplementary Figure 11. FT-IR characterization of i(20)–silica–TPU SMHIP after repeated loading/unloading cycles of a wide range of pressures. Pristine i(20)–silica–TPU SMHIP (spectra are represented in black color), after 5 loading/unloading cycles of ~50 kPa external pressure (spectra are represented in red) and after 5 loading/unloading cycles of ~19 MPa external pressure (spectra are represented in blue). **(a)** Full FT-IR spectra. **(b)** TPU hard segment NH stretching region. **(c)** IL $EMIM^+$ cation stretching region. **(d)** IL $TFSI^-$ anion stretching region. **(e)** TPU carbonyl (C=O) stretching region. Deconvolution of the FTIR peaks was performed by considering peaks Gaussian with a number of iterations to obtain the best-fit Gaussian peak. The ratio of the peak area of H-bonded C=O bands (centers at approximately 1703 cm^{-1} to the free C=O bands (centers at approximately 1730 cm^{-1}) is termed as H-bond index. Error bars represent standard deviation in n = 5 samples. Source data for panel **a-d** are provided as a Source data file.

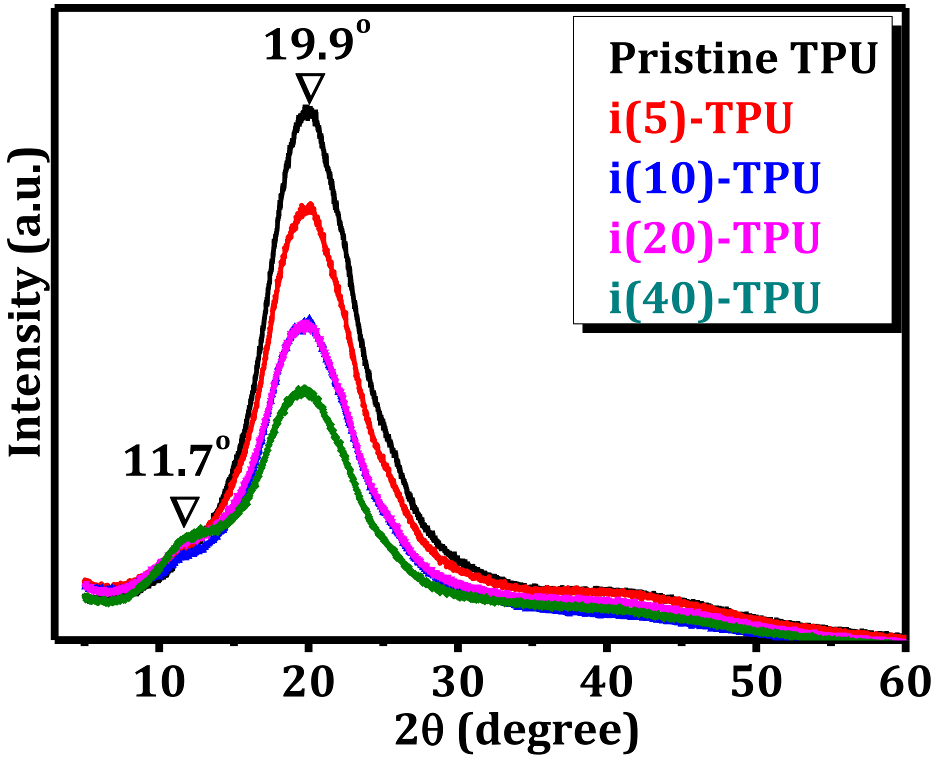

Supplementary Figure 12. XRD patterns of IL–TPU ionic polymer films with varying IL content (5–40 wt%).

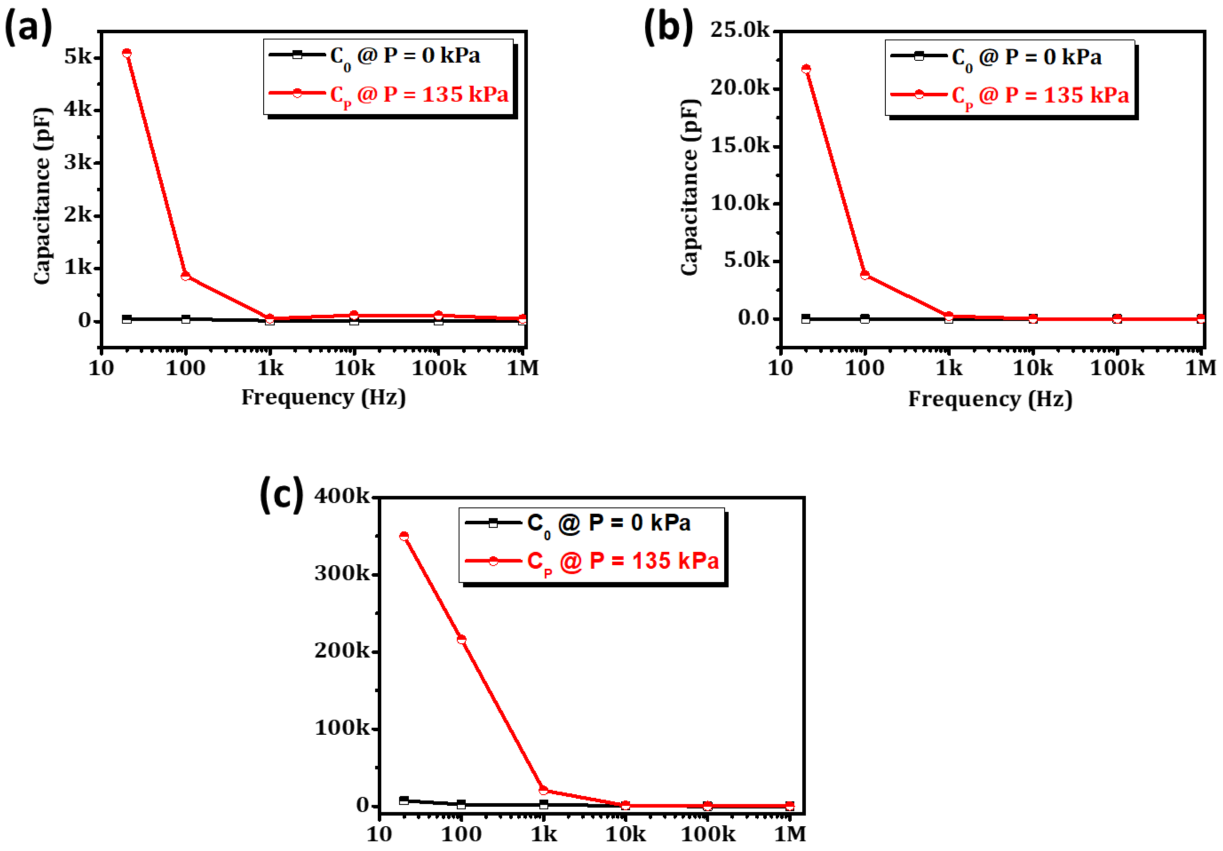

Supplementary Figure 13. Frequency vs. capacitance plots of SMHIP piezocapacitive devices (5–40 wt% IL). **(a)** i(5)–silica–TPU (@ 100 mV). **(b)** i(10)–silica–TPU (@ 1 mV). **(c)** i(40)–silica–TPU (@ 1 mV). C_0 denotes the capacitance without pressure and C_p denotes the capacitance under a pressure of ~135 kPa.

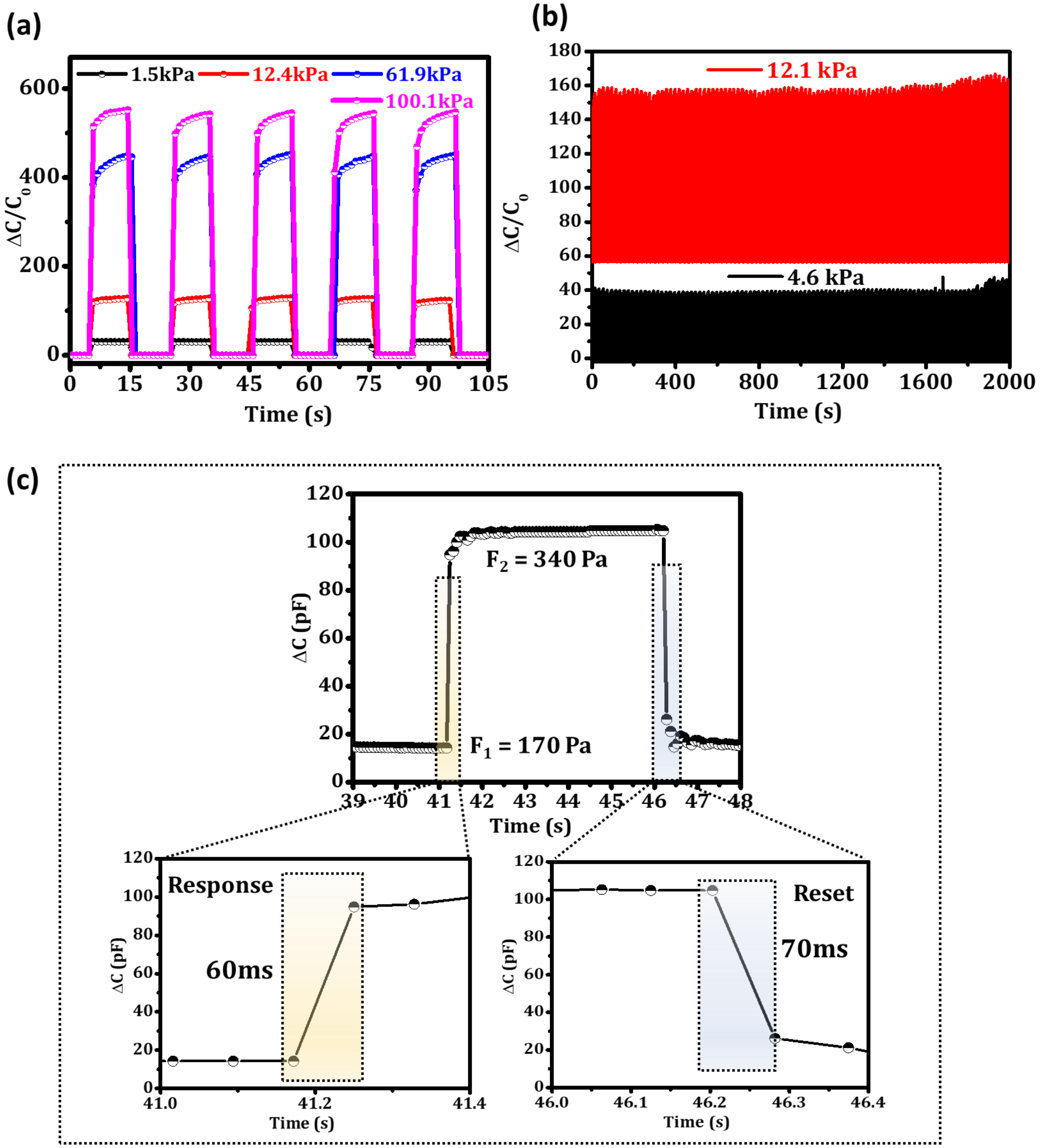

Supplementary Figure 14. Dynamic response of i(20)–silica–TPU SMHIP piezocapacitive pressure sensor. **(a)** Plots of relative change in capacitance ($\Delta C/C_0$) as a function of applied pressure (1.5 kPa, 12.4 kPa, 61.9 kPa and 100.1 kPa, covering a wide pressure-sensing range) as a function of time (applied bias voltage 1V @ 1kHz). **(b)** Durability of the SMHIP-based pressure sensor (500 cycles) under pressure of 4.6 kPa and 12.1 kPa (applied bias voltage 1 V @ 1 kHz). **(c)** Transient response of i(20)–silica–TPU SMHIP piezocapacitive pressure sensor. The inset shows a magnified curve representing a response time of 60 ms and a reset time of 70 ms.

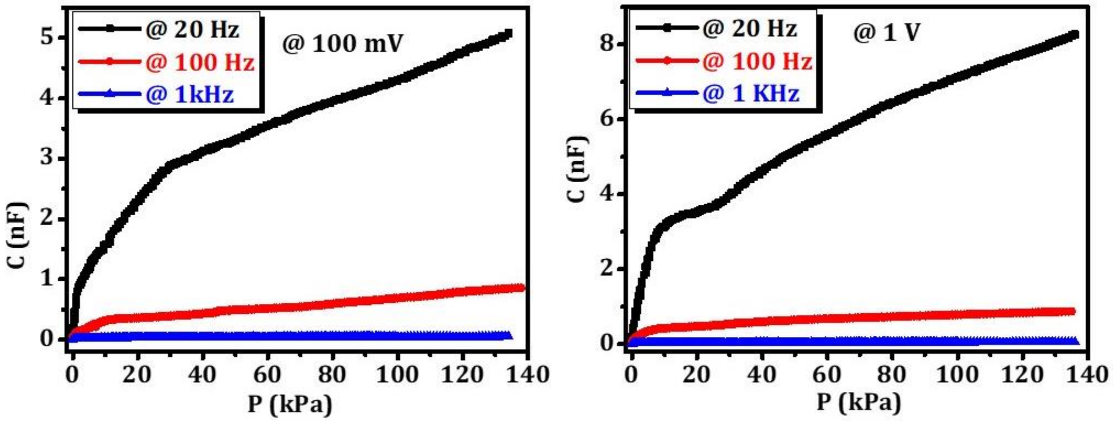

Supplementary Figure 15. Pressure response of i(5)–silica–TPU SMHIP-based piezocapacitive pressure sensors. Capacitance vs. pressure plots under different applied bias voltages (100 mV and 1 V) at various bias frequencies (20 Hz, 100 Hz and 1 kHz). Source data are provided as a Source data file.

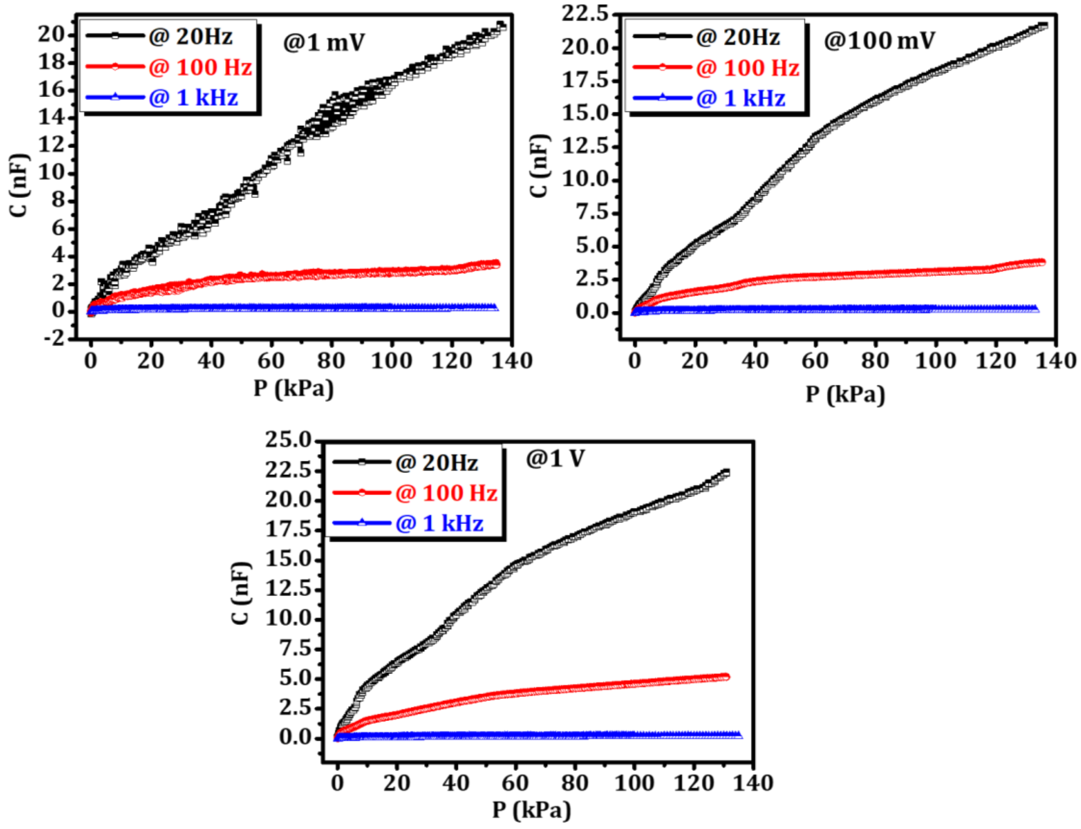

Supplementary Figure 16. Pressure response of i(10)–silica–TPU SMHIP-based piezocapacitive pressure sensors. Capacitance vs. pressure plots under different applied bias voltages (1 mV, 100 mV and 1 V) at various bias frequencies (20 Hz, 100 Hz and 1 kHz). Source data are provided as a Source data file.

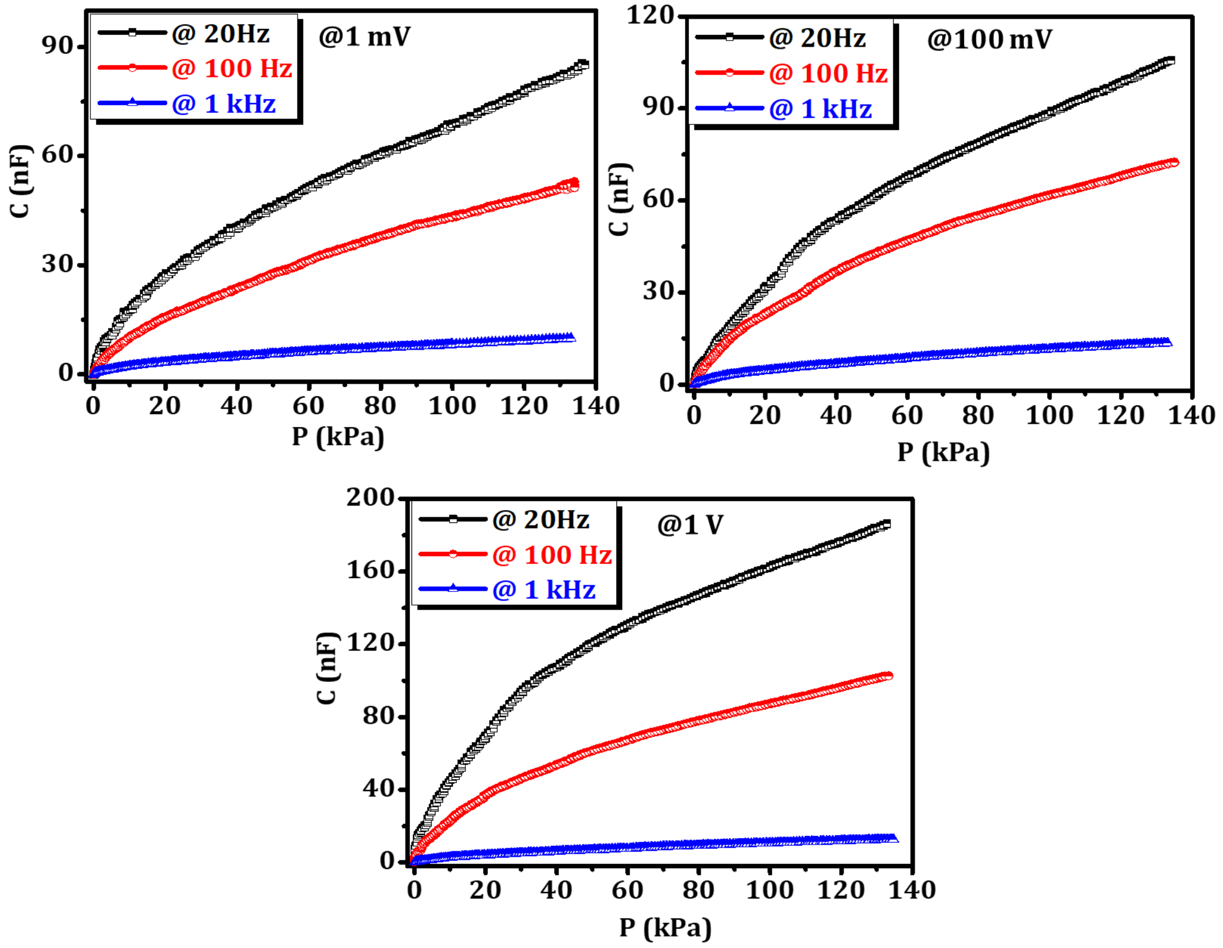

Supplementary Figure 17. Pressure response of i(20)–silica–TPU SMHIP-based piezocapacitive pressure sensors. Capacitance vs. pressure plots under different applied bias voltages (1 mV, 100 mV and 1 V) at various bias frequencies (20 Hz, 100 Hz and 1 kHz). Source data are provided as a Source data file.

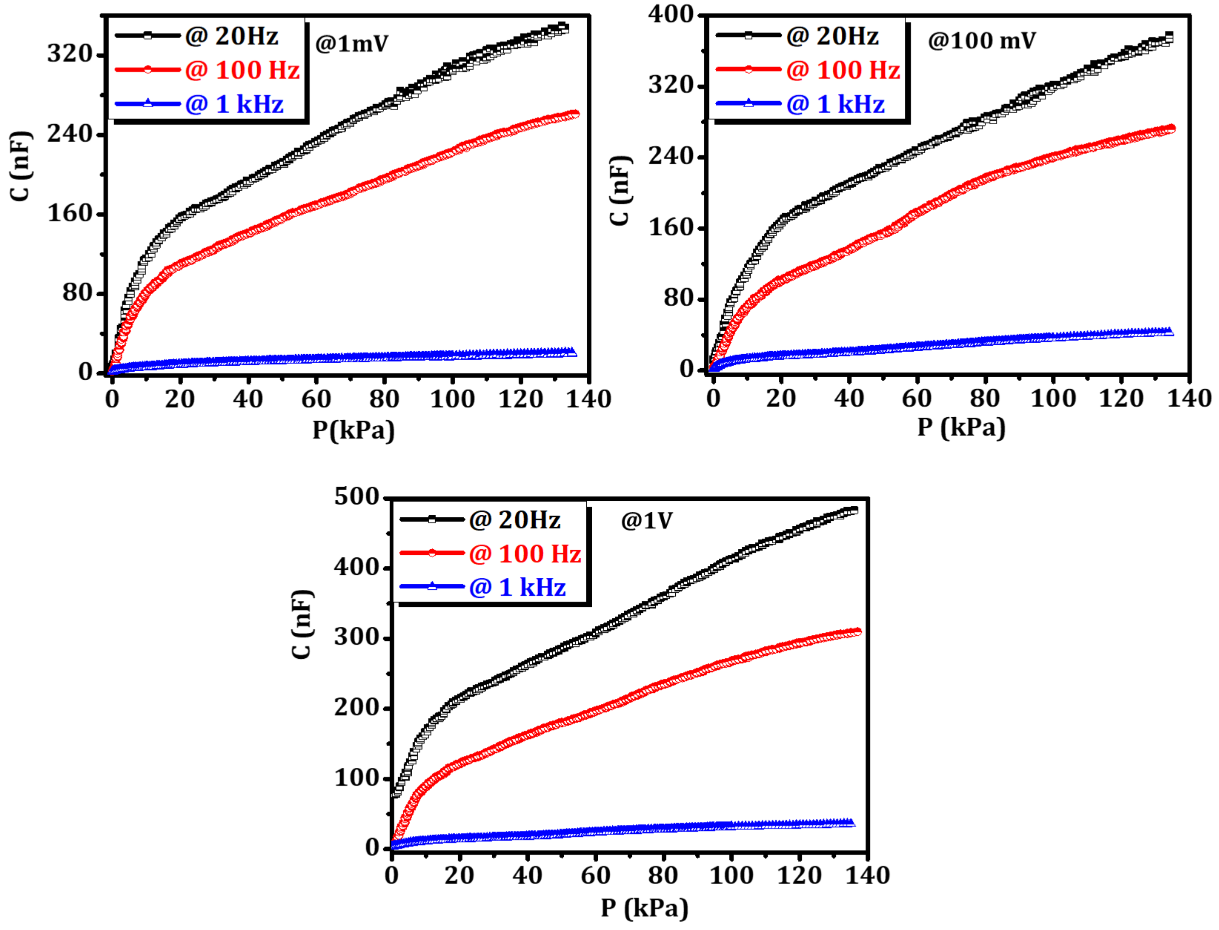

Supplementary Figure 18. Pressure response of i(40)–silica–TPU SMHIP-based piezocapacitive pressure sensors. Capacitance vs. pressure plots under different applied bias voltages (1 mV, 100 mV and 1 V) at various bias frequencies (20 Hz, 100 Hz and 1 kHz). Source data are provided as a Source data file.

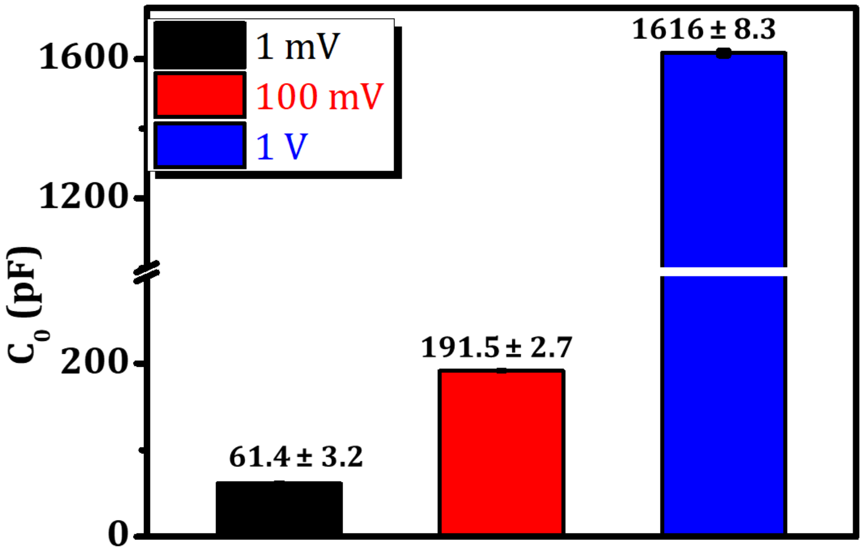

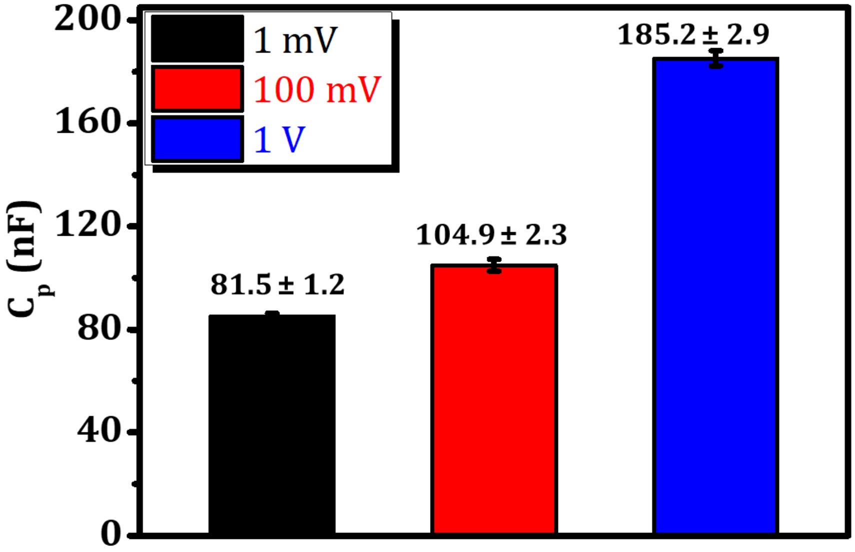

Supplementary Figure 19. Voltage dependent behavior of i(20)–silica–TPU SMHIP-based piezocapacitive devices. Bar graphs represent the initial (C_0, without pressure) and final capacitance (C_p, capacitance under a pressure of ~135 kPa) of i(20)–silica–TPU piezocapacitive pressure sensor (@ 20 Hz).

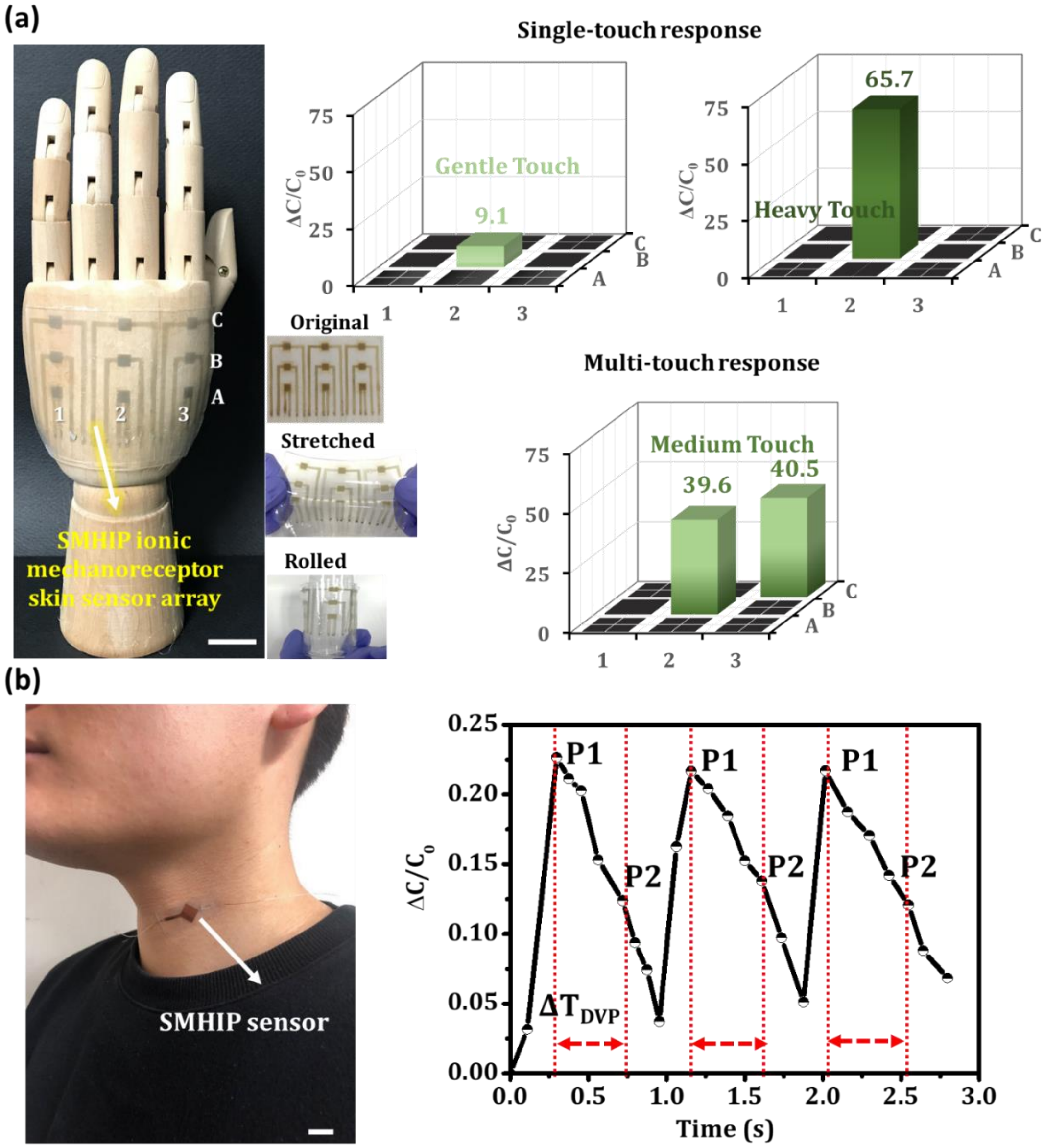

Supplementary Figure 20. Application of SMHIP-based ionic skin sensor array for wearable medical device applications. (a) Photograph of 3 × 3 sensing array of nine pixels (area of each pixel is ~0.25 cm^2, marked by yellow arrow) attached to model hand; Sensing array was fabricated by sandwiching the IL–silica–TPU SMHIP film (film thickness ~170 μm, film area 0.36 cm^2) between two PDMS–Ag nanowires patterned electrodes. Inset demonstrates the excellent stretchability and rollability of our pressure-sensitive ionic skin sensor array. Scale bar, 2 cm. Bar graphs represent on-model hand pressure sensing response of SMHIP-based ionic skin (for details see Supplementary Movie 1). **(b)** Photograph of a SMHIP wearable pressure sensor (marked by white arrow) attached to one of the author's (25 years) neck to record the carotid artery blood pressure waves. Scale bar, 2 cm. Inset is the real-time pulse signal obtained during the experiment.

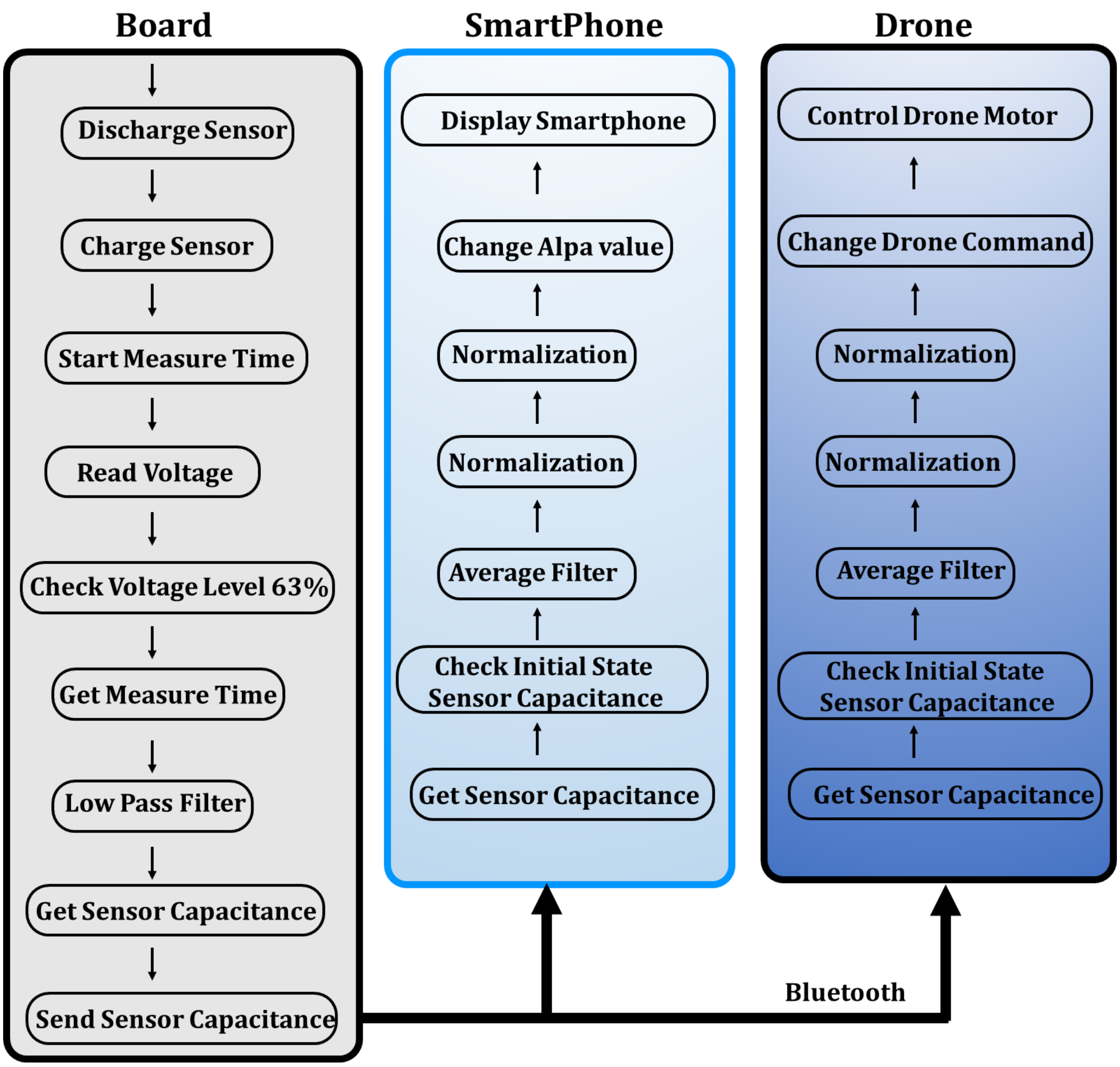

Supplementary Figure 21. A system-level flowchart. Real-time operation of wearable aerial drone microcontroller (WADM) from sensors to custom-developed smartphone application and drone motor. Different steps of signal-conditioning, signal-processing, and wireless transmission from WADM to the custom-developed mobile application and drone controlling application.

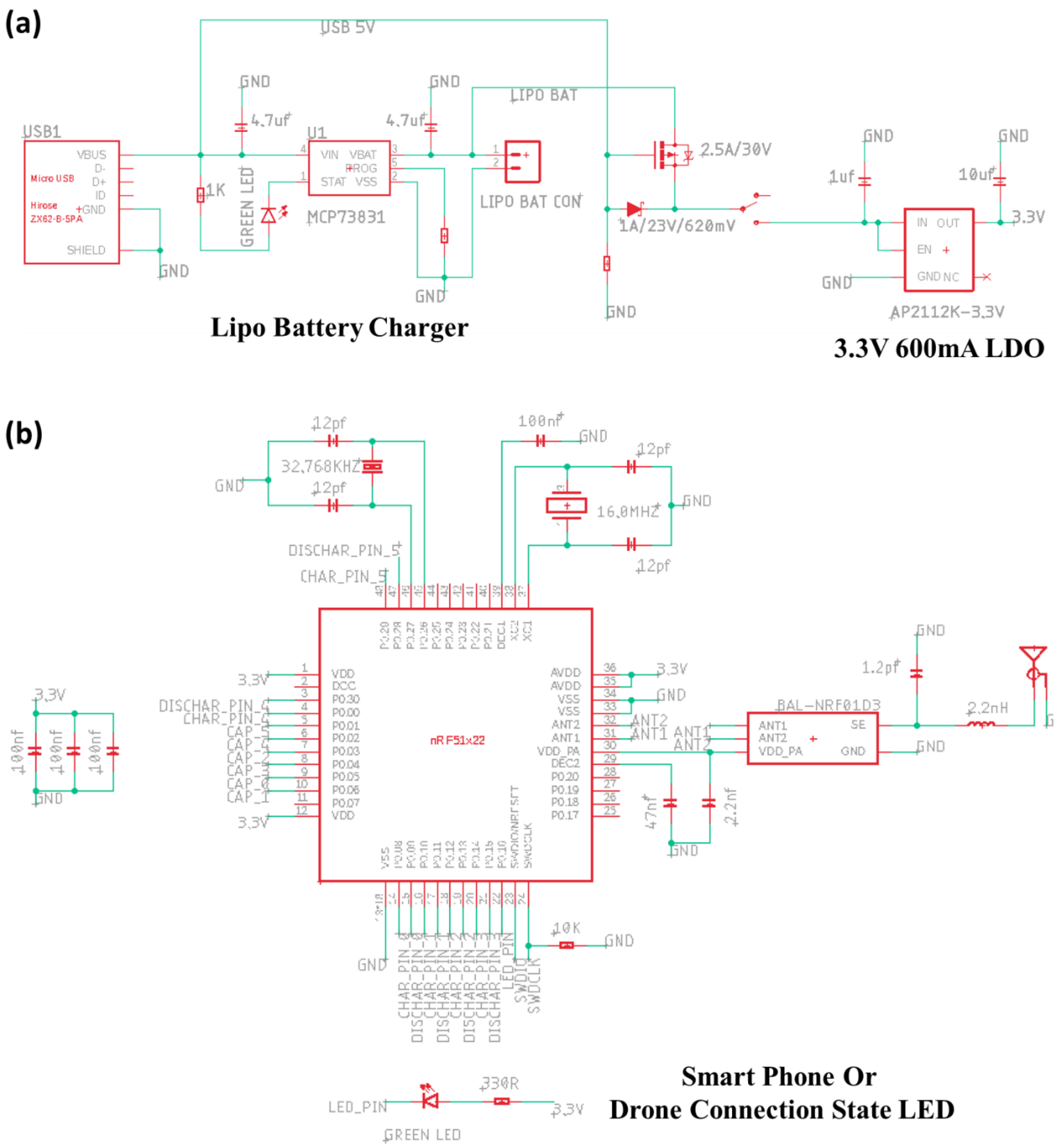

Supplementary Figure 22. Circuit diagram of the signal-conditioning circuit of WADM developed in this work. (a) Battery charge management and controllers. **(b)** Control stage (nRF51822 Bluetooth 4.0 and ARM Cortex M0).

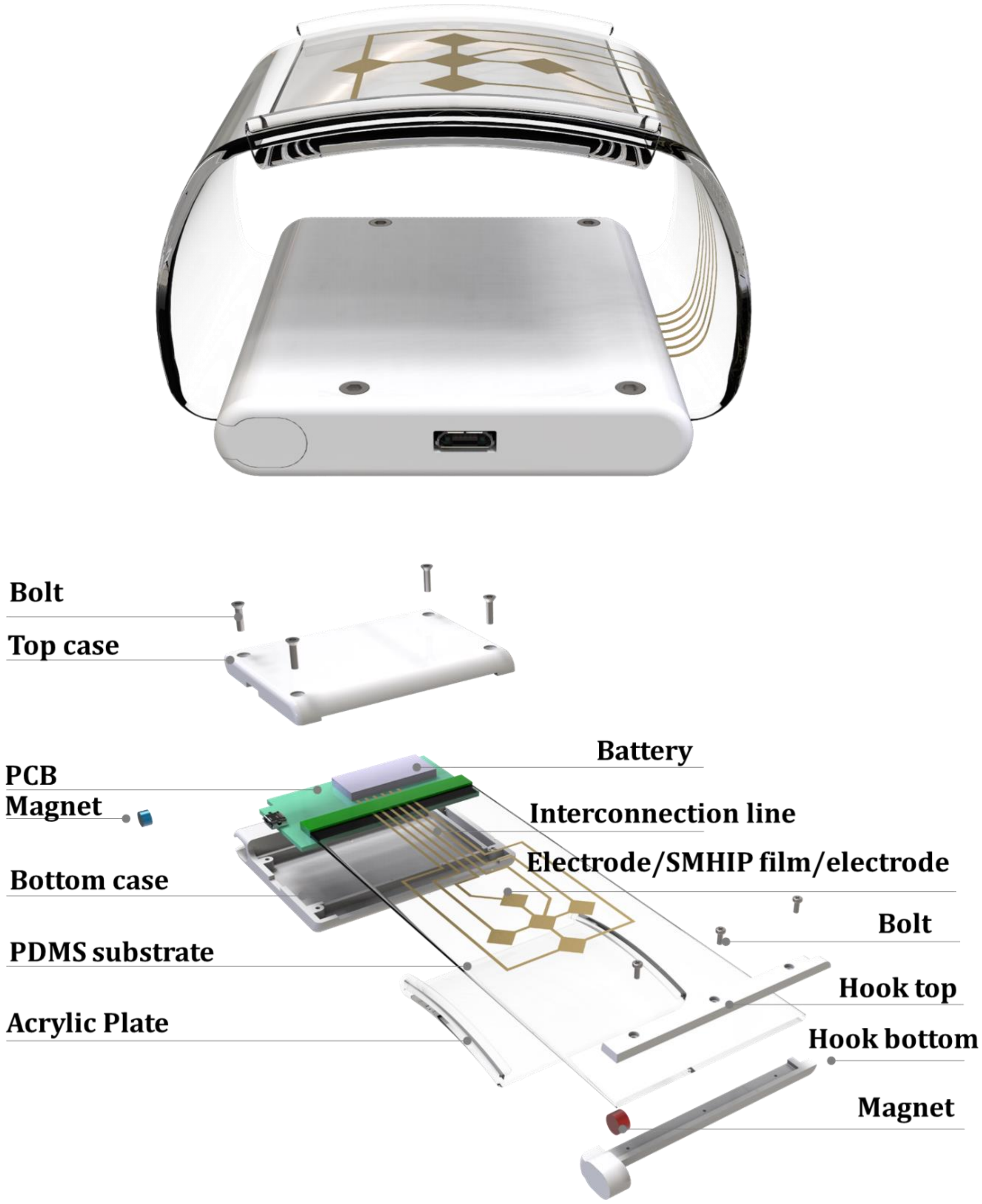

Supplementary Figure 23. Wristband-style WADM for commercial use. Photograph illustrates the hardware components of a wristband-style wearable aerial drone microcontroller.

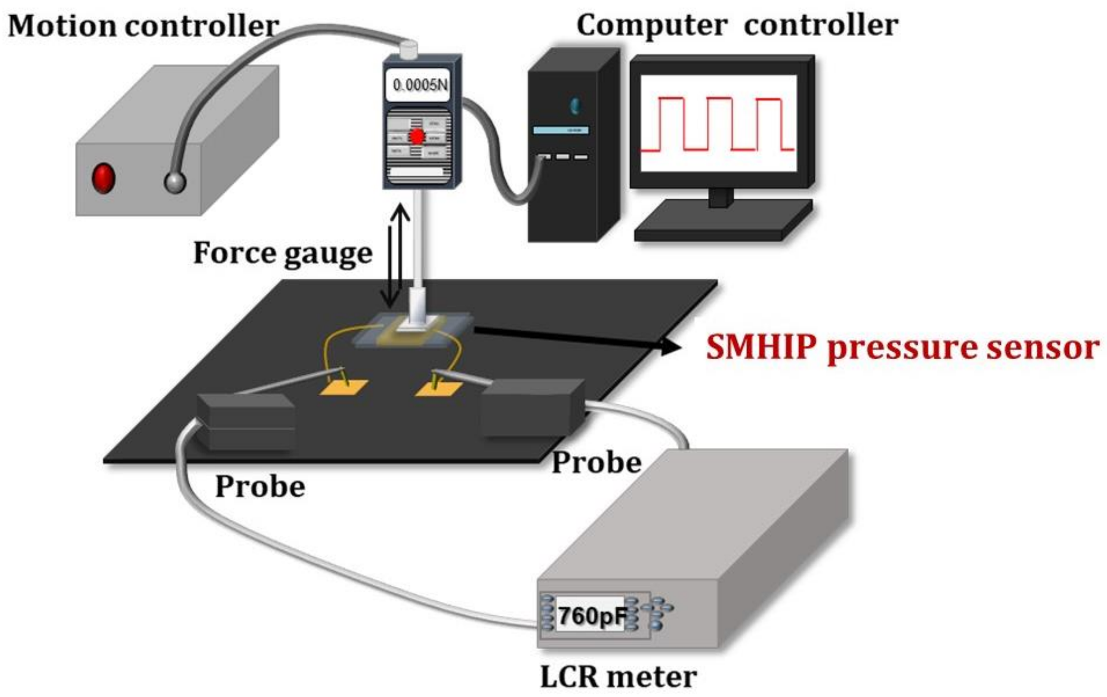

Supplementary Figure 24. Schematic diagram of custom-built pressure sensor probe station. A motion controller and a force gauge are controlled by a computer input with applied pressure showing different value or frequency. The experimental sample is connected with two separated gold electrodes. An LCR meter provides the feedback on the output signal change in capacitance.

Supplementary Table 1. Mechanical properties of pristine TPU polymer, i(20)–TPU ionic polymer, and i(20)–silica–TPU SMHIP films. Experimental results are mean ± standard deviation (obtained from the five samples analyzed under identical experimental conditions).

Sample	Young's modulus (Y) (MPa)	Tensile strength (MPa)	Elongation at the break (ε) (%)
Pristine TPU	19.3 ± 2.9	65.2 ± 3.9	602.2 ± 11.5
i(20)–TPU	10.4 ± 1.4	57.2 ± 1.2	832.8 ± 15.6
i(20)–silica–TPU SMHIP	16.4 ± 1.5	37.4 ± 2.1	686 ± 11.3

Supplementary Table 2. Pressure sensitivity (kPa^{-1}) of i(20)–TPU SMHIP-based pressure sensor (ITO/i(20)–TPU film/ITO) under different experimental conditions.

Biasing Voltage	@ 20 Hz	@ 100 Hz	@ 1 kHz
1 mV	2.55 (0–0.5 kPa)	2.10 (0–0.5 kPa)	0.15 (0–0.5kPa)
	0.58 (2–10 kPa)	0.66 (2–10 kPa)	0.20 (5–20 kPa)
	0.43 (10–30 kPa)	0.61 (20–45 kPa)	0.03 (30–135 kPa)
	0.18 (35–35 kPa)	0.12 (55–135 kPa)	
100 mV	2.12 (0–0.5 kPa)	1.1 (0–0.5 kPa)	0.43 (0–0.5 kPa)
	0.63 (2–13 kPa)	0.61 (2–12 kPa)	0.10 (2–20 kPa)
	0.39 (15–45 kPa)	0.42 (12–40 kPa)	0.05 (30–60 kPa)
	0.13 (60–135 kPa)	0.11 (50–135 kPa)	0.03 (60–135 kPa)
1 V	1.33 (0–0.5 kPa)	1.09 (0–0.5 kPa)	0.35 (0–0.5 kPa)
	0.59 (1–10 kPa)	0.44 (1–10 kPa)	0.33 (1–10 kPa)
	0.05 (10–40 kPa)	0.21 (10–45 kPa)	0.17 (0–35 kPa)
	0.08 (55–135 kPa)	0.05 (45–135kPa)	0.05 (50–135 kPa)

Supplementary Table 3. Pressure sensitivity (kPa^{-1}) of i(20)–silica–TPU SMHIP-based pressure sensor (ITO/i(20)–silica–TPU film/ITO) under different experimental conditions.

Biasing Voltage	@ 20 Hz	@ 100 Hz	@ 1 kHz
1 mV	14.71 (0–0.5 kPa)	48.1 (0–0.5 kPa)	30.86 (0–0.5 kPa)
	24.41 (1–10 kPa)	33.18 (1–5 kPa)	21.10 (1–5 kPa)
	12.33 (10–40 kPa)	14.33 (5–20 kPa)	6.99 (5–10 kPa)
	7.16 (40–135 kPa)	7.72 (20–80 kPa)	2.42 (20–60 kPa)
		5.77 (80–135 kPa)	1.82 (60–135 kPa)
100 mV	31.85 (0–0.5 kPa)	32.48 (0–0.5 kPa)	18.28 (0–0.5 kPa)
	21.5 (0.5–2 kPa)	17.3 (1–5 kPa)	6.61 (1–10 kPa)
	7.14 (5–30 kPa)	14.33 (5–20 kPa)	2.67 (10–20 kPa)
	3.78 (30–60 kPa)	10.23 (20–50 kPa)	2.04 (20–60 kPa)
	2.65 (60–135 kPa)	3.25 (70–135 kPa)	1.35 (60–135 kPa)
1 V	13.43 (0–0.5 kPa)	42.43 (0–0.5kPa)	6.94 (0–0.5kPa)
	1.89 (1–15 kPa)	5.90 (1–20 kPa)	1.61 (1–10 kPa)
	1.46 (15–35 kPa)	2.85 (21–55 kPa)	1.58 (15–40 kPa)
	0.47 (55–135 kPa)	1.82 (60–135 kPa)	0.25 (45-–135 kPa)

Supplementary Table 4. Initial (C_0 @ P = 0 kPa) and final capacitance (C_p @ P = 135 kPa) values of different IL-silica-TPU SMHIP (5–40 wt% IL) piezocapacitive devices (ITO/SMHIP film/ITO, active area ~0.7 cm^2, 1 mV @ 100 Hz). Experimental results are mean ± standard deviation (obtained from the five samples analyzed under identical experimental conditions).

	i(5)-silica-TPU	i(10)-silica-TPU	i(20)-silica-TPU	i(20)-TPU (ref.)	i(40)-silica-TPU
C_0 (pF)	40.8 ± 3.72	28.6 ± 1.4	49.2 ± 3.7	6421 ± 442	3992 ± 66
C_p (nF)	0.86 ± 3.72	3.42 ± 0.32	51.42 ± 2.7	241.98 ± 5.5	272.38 ± 10.9

Supplementary Table 5. Pressure sensitivity (kPa^{-1}) of i(5)–silica–TPU SMHIP-based pressure sensors (ITO/i(5)–silica–TPU active film/ITO) under different experimental conditions.

Biasing Voltage	@ 20 Hz	@ 100 Hz	@ 1 kHz
100 mV	18.2 (< 1 kPa)	0.56 (0–10 kPa)	2.38 (0–5 kPa)
	1.92 (2–30 kPa)	0.34 (10–40 kPa)	1.11 (5–15 kPa)
	0.57 (30–135 kPa)	0.08 (40–135 kPa)	0.10 (40–135 kPa)
	5.36 (60–135 kPa)		
1 V	20.03 (< 1 kPa)	1.68 (< 1 kPa)	0.29 (< 1 kPa)
	1.06 (5–50 kPa)	0.13 (1–50 kPa)	0.04 (1–10 kPa)
	0.25 (50–135 kPa)	0.30 (50–135 kPa)	0.01 (40–135 kPa)

Supplementary Table 6. Pressure sensitivity (kPa^{-1}) of i(10)–silica–TPU SMHIP-based pressure sensors (ITO/i(10)–silica–TPU film/ITO) under different experimental conditions.

Biasing Voltage	@ 20 Hz	@ 100 Hz	@ 1 kHz
100 mV	15.03 (0–10 kPa)	19.16(< 2 kPa)	5.33 (< 2 kPa)
	7.93 (14–35 kPa)	11.5 (2–10 kPa)	1.75 (2–10 kPa)
	12.02 (35–60 kPa)	4.19 (10–30 kPa)	0.44 (10–35 kPa)
	5.36 (60–135 kPa)	1.04 (50–135 kPa)	0.93 (50–135 kPa)
1 V	15.47 (0–2 kPa)	19.09 (< 1 kPa)	10.19 (< 1 kPa)
	8.47 (2–10 kPa)	4.82 (1–10 kPa)	1.39 (1–10 kPa)
	3.95 (10–40 kPa)	2.15 (10–55 kPa)	0.33 (15–40 kPa)
	4.66 (40–60 kPa)	0.87 (55–135 kPa)	0.11 (50–135 kPa)
	2.39 (60–135 kPa)		

Supplementary Table 7. Pressure sensitivity (kPa^{-1}) of i(40)–silica–TPU SMHIP-based pressure sensors (ITO/i(40)–silica–TPU film/ITO) under different experimental conditions.

Biasing Voltage	@ 20 Hz	@ 100 Hz	@ 1 kHz
1 mV	2.1 (0–5 kPa)	5.16 (0–3 kPa)	5.33 (0–2 kPa)
	0.77 (5–15 kPa)	11.5 (3–10 kPa)	1.75 (2–10 kPa)
	0.27 (20–75 kPa)	4.19 (10–30 kPa)	0.44 (10–35 kPa)
	7.16 (90–135 kPa)	1.04 (50–135 kPa)	0.09 (50–135 kPa)
100 mV	1.02 (0–5 kPa)	1.86 (0–5 kPa)	1.03 (0–2 kPa)
	0.42 (5–20 kPa)	0.44 (15–50 kPa)	0.12 (5–18 kPa)
	0.14 (20–135 kPa)	0.53 (50–75 kPa)	0.14 (30–90 kPa)
		0.26 (80–135 kPa)	0.09 (90–135 kPa)
1 V	0.12 (0–8 kPa)	1.38 (0–10 kPa)	0.28 (0–5 kPa)
	0.04 (10–18 kPa)	0.40 (10–22 kPa)	0.05 (15–45 kPa)
	0.03 (20–80 kPa)	0.25 (20–75 kPa)	0.08 (50–70 kPa)
	0.33 (80–135 kPa)	0.21 (80–135 kPa)	0.05 (90–135 kPa)

Supplementary Table 8. Comparison of the pressure sensitivity and the operating range of the pressure sensors obtained in this work with previously reported pressure sensors.

Device Type	Materials	Sensitivity (kPa^{-1})	Range	References
Piezocapacitive	PAM–NaCl/acrylic elastomer	0.01 (0–40 kPa)	0–40 kPa	22
Piezoresistive	Pt–coated polyurethane–based nanofibres	11.45 (~5 Pa)	0.005–1.5 kPa	24
Piezoresistive	PDMS/metal–coated hierarchical ZnO NW arrays	6.8 (< 0.3 kPa)	0.0006–10 kPa	25
Piezocapacitive/ organic thin-film pressure sensor	Microstructured PDMS dielectric	0.55 (< 2 kPa)<br>0.15 (> 2 kPa)	0–7 kPa	26
Piezoresistive	Polypyrrole/ITO–PET	56.0–133.1 (< 0.03 kPa)<br>0.4 (> 1kPa)	0.01–10 kPa	27
Piezocapacitive	CNT–Ecoflex/Porous Ecoflex	0.601 (<5 kPa)<br>0.077 (30–130 kPa)	0–130 kPa	28
Organic thin-film transistor type	PET–PDPP3T and CYTOPPI (semiconductor)/Al foil (suspended gate)	192 (< 5 kPa)	0–50 kPa	29
Organic thin-film transistor type	ITO–PET/Microstructured PDMS/	8.4 (< 2 kPa)<br>0.38 (10–50 kPa)	0–50 kPa	30
Piezoresistive	PVDF electrospun yarns of nanofibers coated with PEDOT	18.37 (~0.1 kPa)	0–12 kPa	31
Piezoresistive	Pt–PDMS/MWCNT–PDMS	15.1 (< 0.5 kPa)	0–60 kPa	32
Piezoresistive	Ultralight Sparkling-Graphene Block	229.8 (0–0.1 kPa)<br>26.86 (0.4–1 kPa)	0.02–1.2 kPa	33
Piezoresistive	Polystyrene ball@reduced graphene–oxide core–shell nanoparticles	50.9 (0.003–1 kPa)	0–20 kPa	34

Piezocapacitive	ITO/IL–silica–TPU	48.1–33.18 (0–5 kPa) 5.77–7.16 (80–135 kPa)	0 –135 kPa	Our work

Supplementary Table 9. Comparison of the operating voltage and the achieved pressure sensitivity of the pressure sensors obtained in this work with previously reported pressure sensors.

Device type	Materials	Sensitivity (kPa^{-1})	Operating voltage	References
Piezocapacitive	PAM–NaCl/acrylic elastomer	0.01 (0 –40 kPa)	1 V	22
Piezocapacitive/ organic thin-film pressure sensor	Microstructured PDMS dielectric	0.55 (< 2 kPa) 0.15 (> 2 kPa)	20 V	26
Organic thin-film transistor type	PET–PDPP3T and CYTOPPI (semiconductor)/Al foil (suspended gate)	192 (< 5 kPa)	60 V	29
Piezoresistive	Pt–PDMS/MWCNT–PDMS	15.1 (< 0.5 kPa)	10 V	32
Piezoresistive	Ultralight Sparkling-Graphene Block	229.8 (0–0.1 kPa) 26.86 (0.4–1 kPa)	0.1 V	33
Piezoresistive	Polystyrene ball@reduced graphene–oxide core–shell nanoparticles	50.9 (0.003–1 kPa)	1 V	34
Piezoresistive	SWNTs/PDMS	1.80 (~ 0.6 Pa)	2 V	35
Piezocapacitive	Microstructured PDMS	0.815 (0–50 N)	1 V	36
Piezoresistive	Au/Microdomed PDMS	15 (0–100 Pa)	0.1 V	37
Piezoresistive	AuNWs–impregnated tissue paper	1.14 (0–5 kPa)	1.5 V	38
Piezoresistive	polyaniline (PANi)–polyvinylidene difluoride (PVDF)/ polycarbonate ion channel pressure sensors	5.6 (0.2–1 kPa)	0.1 V	39

Piezocapacitive	ITO/IL–silica–TPU	48.1– 33.18 (0–5 kPa) 5.77–7.16 (80–135 kPa)	1 mV	Our work

Supplementary References

1. Jin, M. L. et al. An ultrasensitive, visco-poroelastic artificial mechanotransducer skin inspired by Piezo2 protein in mammalian Merkel cells, *Adv. Mater*. **29**, 1605973 (2017).

2. Nayeri, M. et al. Surface effects on the structure and mobility of the ionic liquid C_6C_1ImTFSI in silica gels. *Soft Matter*, **10**, 5618–5627 (2014).

3. Néouze, M-A. et al. Ionogels, New materials arising from the confinement of ionic liquids within silica-derived networks. *Chem. Mater.* **18**, 3931–3936 (2006).

4. Wang, T. et al. Electroactive polymers for sensing. *Interface Focus* **6**, 20160026 (2016).

5. Hong, W., Almomani, A., Chen, Y., Jamshidi, R. and Montazami, R. Soft ionic electroactive polymer actuators with tunable non-linear angular deformation, *Materials* **10**, 664 (2017).

6. Voorhaar, L., Hoogenboom, R. Supramolecular polymer networks: hydrogels and bulk materials, *Chem. Soc. Rev.* **45**, 4013–4031 (2016).

7. Burattini, S. et al. A healable supramolecular polymer blend based on aromatic $\pi-\pi$ stacking and hydrogen-bonding interactions. *J. Am. Chem. Soc.* **132**, 34, 12051–12058 (2010).

8. Wei, Z. et al. Self-healing gels based on constitutional dynamic chemistry and their potential applications. *Chem. Soc. Rev.* **43**, 8114–8131 (2014).

9. Guo, Y., Zhou, X., Tang, Q., Bao, H., Wang, G. and Saha, P. A self-healable and easily recyclable supramolecular hydrogel electrolyte for flexible supercapacitors. *J. Mater. Chem. A* **4**, 8769–8776 (2016).

10. Hoffmann, F., Cornelius, M., Morell, J., Fröba, M. Silica-based mesoporous organic–inorganic hybrid materials. *Angew. Chem. Int. Ed*. **45**, 3216-3251 (2006).

11. Brinker, C.J. Hydrolysis and condensation of silicates: effects on structure. *J. Non-Cryst. Solids* **100**, 31–50 (1988).

12. Lindberg, R., Sjoblom, J., Sundholm, G. Preparation of silica particles utilizing the sol-gel and the emulsion-gel processes. *Colloids and Surfaces A: Physicochemical and Engineering Aspects*. **99**, 79–88 (1995).

13. Verma, Y.L., Singh, R. K. Conformational states of ionic liquid 1-Ethyl-3-methylimidazolium Bis(trifluoromethylsulfonyl)imide in bulk and confined silica nanopores probed by crystallization kinetics study. *J. Phys. Chem. C* **119**, 24381–24392 (2015).

14. Donato, K. Z., Matejka L., Mauler, R. S., & Donato, R. K. Recent applications of ionic liquids in the sol-gel process for polymer–silica nanocomposites with ionic interfaces. *Colloids Interfaces*, **1**, 1–25 (2017).

15. Donato, R.K., Benvegnú, M.A., Furlan, L.G., Mauler, R.S., Schrekker, H.S. Imidazolium salts as liquid coupling agents for the preparation of polypropylene-silica composites. *J. Appl. Polym. Sci.* **116**, 304–307 (2010).

16. Ma, Z. Yu, J. & Dai, S. Preparation of inorganic materials using ionic liquids. *Adv. Mater.* **22**, 261–285 (2010).

17. Li, S., et al. Dynamic and structural properties of room-temperature ionic liquids near silica and carbon surfaces. Langmuir **29**, 9744–9749 (2013).

18. Magonov, S. N., Vainilovitch, I. S., & Sheiko, S. S. FTIR spectroscopy of polymer films under uniaxial stretching. *Polym. Bull.* **25**, 491, 491–498 (1991).

19. Zadler, L. et al. Monitoring the chemistry of self-healing by vibrational spectroscopy – current state and perspectives. *Mater. Today* **17**, 57-69 (2014).

20. Werling, K. A., Hutchison, G.R., & Lambrecht, D.S. Piezoelectric effects of applied electric fields on hydrogen-bond interactions: first-principles electronic structure investigation of weak electrostatic interactions. *J. Phys. Chem. Lett.* **4**, 1365–1370 (2013).

21. James, H. P., John, R., Alex, A., & Anoop, K.R. Smart polymers for the controlled delivery of drugs – a concise overview. *Acta Pharmaceutica Sinica B* **4**, 120–127 (2014).

22. Sun, J-Y., Keplinger C., Whitesides G. M., & Suo, Z. Ionic skin. *Adv. Mater*. **26**, 7608–7614 (2014).

23. Pang, C. et al. A flexible and highly sensitive strain-gauge sensor using reversible interlocking of nanofibres. *Nat. Mater.* **11**, 795–801 (2012).

24. Ha, M. et al. Bioinspired interlocked and hierarchical design of ZnO nanowire arrays for static and dynamic pressure-sensitive electronic skins. *Adv. Funct. Mater*. **25**, 2841–2849 (2015).

25. Kang, S. et al. Highly sensitive pressure sensor based on bioinspired porous structure for real-time tactile sensing. *Adv. Electron. Mater*. **2**, 1600356 (2016).

26. Mannsfeld, S. C. B. et al. Highly sensitive flexible pressure sensors with microstructured rubber dielectric layers. *Nature Mater*. **9**, 859–864 (2010).

27. Pan, L. et al. An ultra-sensitive resistive pressure sensor based on hollow-sphere microstructure induced elasticity in conducting polymer film. *Nat. Commun.* **5**, 3002 (2014).

28. Kwon, D. et al. Highly sensitive, flexible, and wearable pressure sensor based on a giant piezocapacitive effect of three-dimensional microporous elastomeric dielectric layer. *ACS Appl. Mater. Interfaces* **8**, 16922–16931 (2016).

29. Zang, Y. et al. Flexible suspended gate organic thin-film transistors for ultra-sensitive pressure detection. *Nat. Commun.* **6**, 6269, (2015).

30. Schwartz, G. et al. Flexible polymer transistors with high pressure sensitivity for application in electronic skin and health monitoring. *Nat. Commun.* **4**, 1859 (2013).

31. Zhou, Y. et al. Highly sensitive, self-powered and wearable electronic skin based on pressure-sensitive nanofiber woven fabric sensor. *Sci Rep.* **7**, 12949 (2017).

32. Park, J. et al. Giant tunneling piezoresistance of composite elastomers with interlocked microdome arrays for ultrasensitive and multimodal electronic skins. *ACS Nano* **8**, 4689–4697 (2014).

33. Lv, L., Zhang, P., Xu, T. & Qu, L. Ultrasensitive pressure sensor based on an ultralight sparkling graphene bock. *ACS Appl. Mater. Interfaces* **9**, 22885–22892 (2017).

34. Ai, Y. et al. An ultrasensitive flexible pressure sensor for multimodal wearable electronic skins based on large-scale polystyrene ball@reduced graphene-oxide core–shell nanoparticles. *J. Mater. Chem. C* **6**, 5514–5520 (2018).

35. Wang, X., Gu, Y., Xiong, Z., Cui, Z., & Ting, Z. Silk-molded flexible, ultrasensitive, and highly stable electronic skin for monitoring human physiological signals. *Adv. Mater.* **26**, 1336–1342 (2014).

36. Li, T. et al. Flexible capacitive tactile sensor based on micropatterned dielectric layer. *Small* **12**, 5042–048 (2016).

37. Zhang, Y. et al. Flexible and highly sensitive pressure sensor based on microdome-patterned PDMS forming with assistance of colloid self-assembly and replica technique for wearable electronics. *ACS Appl. Mater. Interfaces* **9**, 35968–35976 (2017).

38. Gong, S. et al. A wearable and highly sensitive pressure sensor with ultrathin gold nanowires. *Nature Commun.* **5**, 3132 (2014).

39. Chun, K-Y., Son, Y. J. & Han, C-S. Highly sensitive and patchable pressure sensors mimicking ion-channel-engaged sensory organs. *ACS Nano* **10**, 4550–4558 (2016).
